# Comparative proteomic analysis of *Neisseria meningitidis* wildtype and *dprA* null mutant strains links DNA processing to pilus biogenesis

**DOI:** 10.1186/s12866-017-1004-8

**Published:** 2017-04-21

**Authors:** Getachew Tesfaye Beyene, Shewit Kalayou, Tahira Riaz, Tone Tonjum

**Affiliations:** 1 Department of Microbiology, University of Oslo, Oslo, Norway; 2 Department of Microbiology, Oslo University Hospital, Oslo, Norway; 3 Mekelle University College of Veterinary Medicine, Mekelle, Ethiopia; 4 Present address: College of Health Sciences, Mekelle University, Mekelle, Ethiopia

**Keywords:** *Neisseria meningitidis*, DNA processing, Pilus biogenesis, DprA, PilG, Proteomics, Mass spectrometry

## Abstract

**Background:**

DNA processing chain A (DprA) is a DNA binding protein which is ubiquitous in bacteria, and is required for DNA transformation to various extents among bacterial species. However, the interaction of DprA with competence and recombination proteins is poorly understood. Therefore, the proteomes of whole *Neisseria meningitidis* (Nm) wildtype and *dprA* mutant cells were compared. Such a comparative proteomic analysis increases our understanding of the interactions of DprA with other Nm components and may elucidate its potential role beyond DNA processing in transformation.

**Results:**

Using label-free quantitative proteomics, a total of 1057 unique Nm proteins were identified, out of which 100 were quantified as differentially abundant (*P ≤ 0.05* and fold change ≥ |2|) in the *dprA* null mutant. Proteins involved in homologous recombination (RecA, UvrD and HolA), pilus biogenesis (PilG, PilT1, PilT2, PilM, PilO, PilQ, PilF and PilE), cell division, including core energy metabolism, and response to oxidative stress were downregulated in the Nm *dprA* null mutant. The mass spectrometry data are available via ProteomeXchange with identifier PXD006121. Immunoblotting and co-immunoprecipitation were employed to validate the association of DprA with PilG. The analysis revealed reduced amounts of PilG in the *dprA* null mutant and reduced amounts of DprA in the Nm *pilG* null mutant. Moreover, a number of pilus biogenesis proteins were shown to interact with DprA and /or PilG.

**Conclusions:**

DprA interacts with proteins essential for Nm DNA recombination in transformation, pilus biogenesis, and other functions associated with the inner membrane. Inverse downregulation of Nm DprA and PilG expression in the corresponding mutants indicates a link between DNA processing and pilus biogenesis.

**Electronic supplementary material:**

The online version of this article (doi:10.1186/s12866-017-1004-8) contains supplementary material, which is available to authorized users.

## Background

*Neisseria meningitidis* (Nm) is a human commensal and pathogen that in the lack of bactericidal antibodies may cause meningitis and/or septicaemia [1]. Nm has a small (~ 2.2 Mb) and hyperdynamic genome. The pathogenic *Neisseria* species, Nm and *N. gonorrhoeae* (Ng) are naturally and constitutively competent for uptake of exogenous DNA provided that they express type 4 pili (Tfp), can perform RecA-dependent recombination, and find the abundantly occurring DNA uptake sequence (DUS) in the transforming DNA [2–4]. Transformation is the main form of horizontal gene transfer (HGT) in *Neisseria sp*., enabling these species to generate extensive genetic diversity [5, 6].

During transformation, the incoming DNA is processed by RecA [7, 8], DNA processing chain A (DprA), recombination mediator protein (RMP), and single-stranded DNA-binding protein (SSB) [4, 9–13]. The association of RecA with DNA is mainly its central role in homologous recombination [14]. DprA from *Streptococcus pneumoniae*, *Bacillus subtilis,* and *Helicobacter pylori* was shown to take part in intracellular DNA processing, interact with RecA, and displace SSB from ssDNA [15, 16]. In addition, DprA loads RecA onto ssDNA, promoting annealing of homologous ssDNA, and protects incoming DNA [15–18]. The DprA and RecA proteins bind strongly and in long clusters to ssDNA to form a nucleoprotein filament [15, 17]. DprA selectively binds and protects ssDNA from nucleases [8]. DprA plays a role in transformation in all bacterial species examined except for *Escherichia coli*; however, the transformability of *dprA* null mutants varies among bacterial species and DNA substrates. Using a transposon mutant screen in Nm, Tang and co-workers showed that the *dprA* null mutant exhibits total loss of competence for DNA transformation [4]. The Nm and Ng *dprA* null mutants are non-transformable regardless of the type of donor DNA substrate, and Ng DprA is suggested to be involved in RecA-mediated pilin variation [4, 19]. HGT in *Haemophilus influenzae, S. pneumoniae*, and *B. subtilis* is associated with DprA [8, 15, 20, 21]. DprA in *S. pneumoniae* is involved in an intracellular signalling cascade that turns off competence [22, 23]. DprA in *B. subtilis* appears to increase the efficiency of RecA strand exchange during transformation and forms a large multiprotein complex with RecA, SSB-B and other competence proteins [17, 24].

Here, we performed a comprehensive proteomic analysis of Nm wildtype and *dprA* null mutant cells to define their protein profile and to search for interactions between DprA and other Nm components. For this purpose, the cell lysates from the Nm wildtype and *dprA* null mutant strains were prepared, and the proteins were subjected to in-gel digestion. The resulting peptide products were subsequently analysed by using high resolution mass spectrometry (MS).

In this global quantitative proteomic analysis, multiple proteins identified were significantly less abundant in the *dprA* null mutant including those involved in Tfp biogenesis, recombination, cell division and energy metabolism. A link between DprA and the inner membrane protein PilG and other pilus biogenesis proteins was thereby detected. Immunoblotting and co-immunoprecipitation (Co-IP) were employed to validate the interaction between DprA and PilG. In general, these findings elucidate the role of DprA in Nm cells and its interaction with components of the transformation, Tfp biogenesis, and other machineries.

## Results

### Predominantly less abundant proteins detected in the Nm Δ*dprA* mutant

**Fig. 1:**
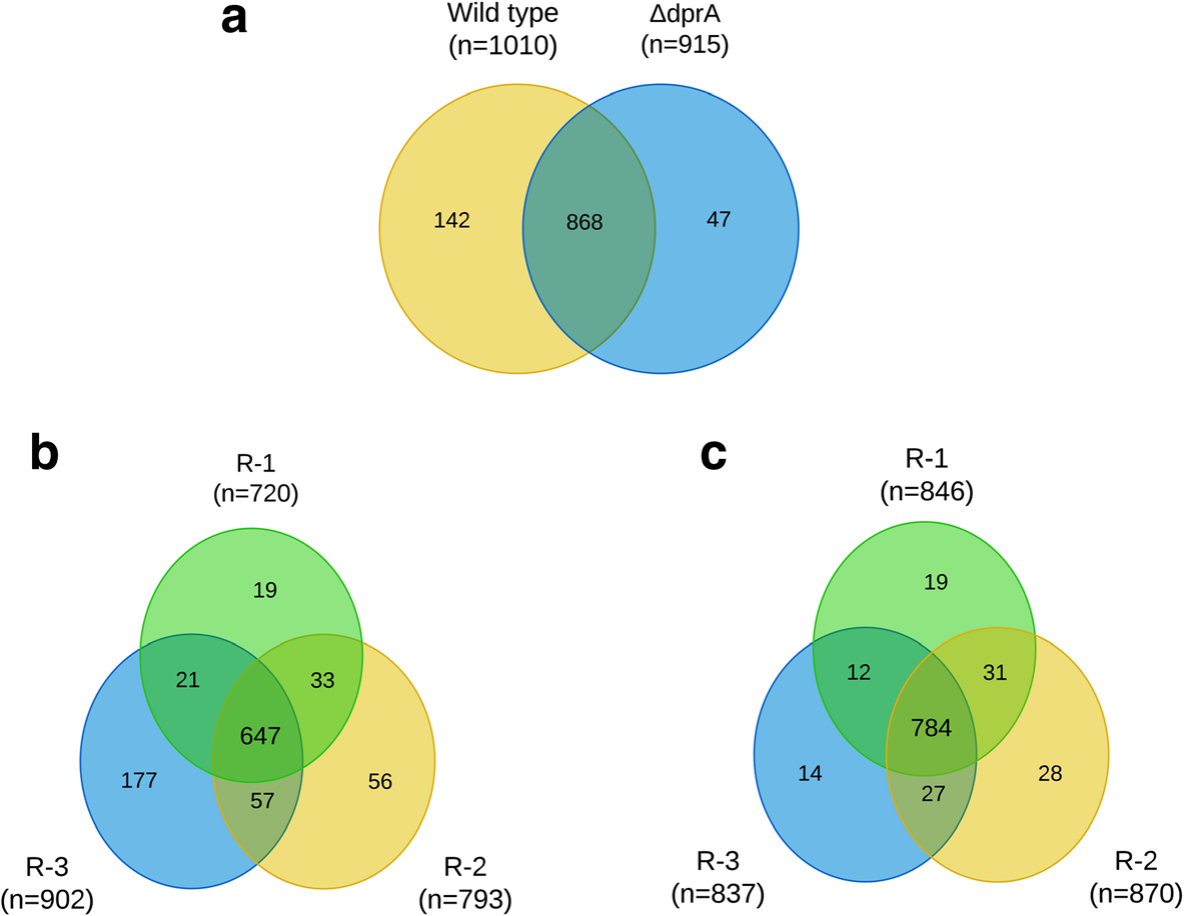
Venn plot illustrating protein identification overlaps between wildtype and Δ*dprA* (**a**) and among the three biological replicates (R1–R3) in *ΔdprA* mutant (**b**) and Wildtype (**c**)

To assess DprA-associated changes in the Nm proteome, a quantitative analysis of Nm wildtype and *dprA* mutant strains was conducted by applying a liquid chromatography tandem mass spectrometry (LC-MS/MS)-based label free quantitative (LFQ) proteomics approach. Total soluble lysate from three biological replicates were separated by one dimensional (1D) SDS-PAGE. After tryptic in-gel digestion, six gel fractions from each replicate were analyzed by high performance liquid chromatography (HPLC) coupled with Q Exactive MS in technical triplicates. This workflow generated a total of 108 Raw MS files. The resulting data was analyzed together in the MaxQuant environment specifying a confidence rate of 99% at the peptide and protein level. This identified a total of 1057 protein groups, with 1010 proteins identified in the wildtype, and 915 proteins identified in the DprA null mutant (Fig. 1a, Additional file 1: Table S1). Analysis for overlap of protein identification showed an overlap of 70.7% (647/915) and 77.6% (784/1010) protein identification across all the biological experiments in the *dprA* mutant and wildtype, respectively (Fig. 1a-c). Likewise, the overall overlap between *dprA* mutant and wildtype samples was 82% (868/1057). In order to check the reproducibility of our label-free quantification workflow, we computed the Pearson correlations (R values) of biological replicates using normalized protein LFQ intensities. The analysis showed that the R values (range 0.67–0.96) between normalized intensities were high and was thus suited for accurate comparisons of protein abundance differences.

**Fig. 2:**
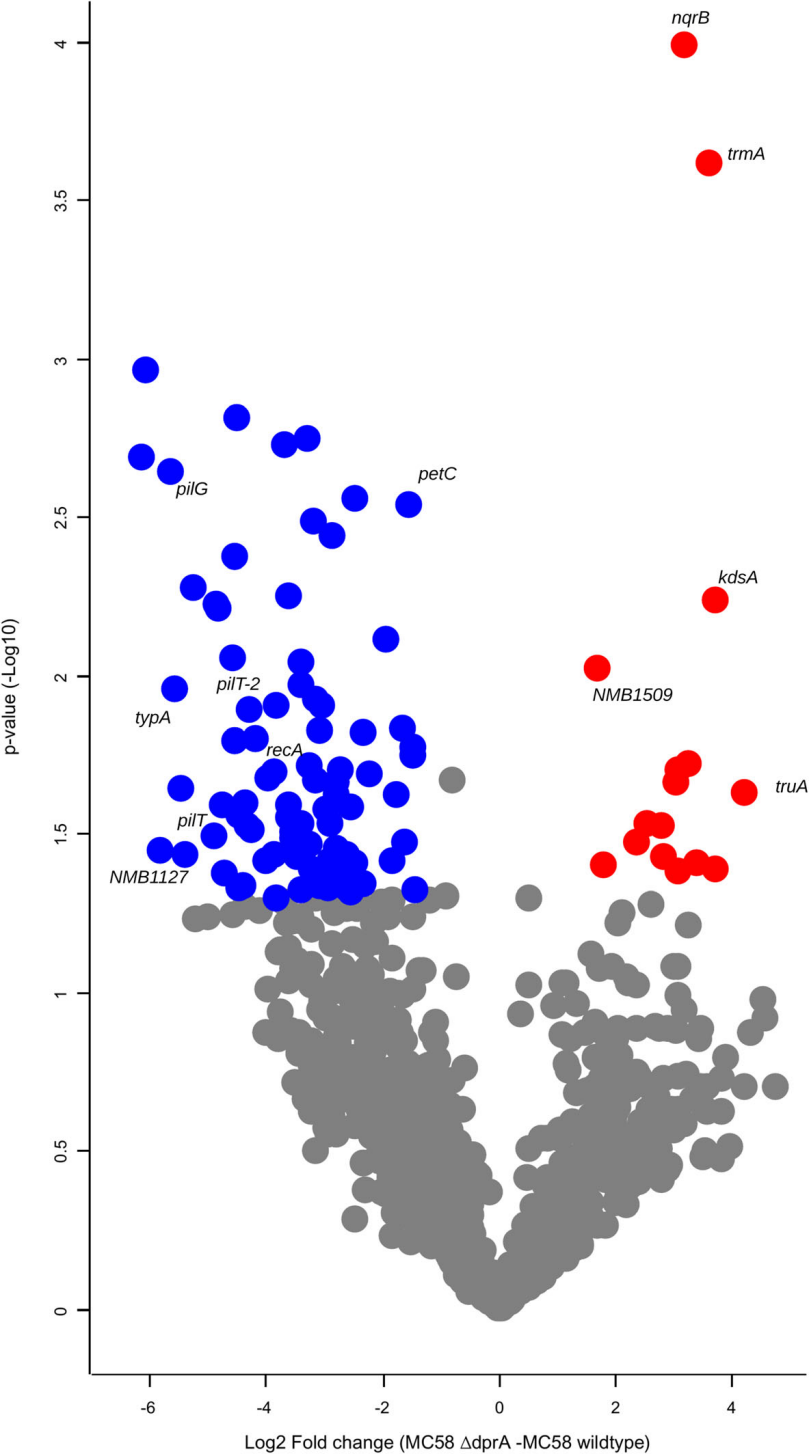
Quantitative analysis of differential protein abundances in Nm serotype B. Volcano plot of protein abundance differences as a function of statistical significance (t test *p* ≤ 0.05 and fold change cutoff ≥2) between *dprA* null mutant and wildtype Nm. More abundant proteins are indicated by blue dots and less abundant proteins by red dots

**Fig. 3:**
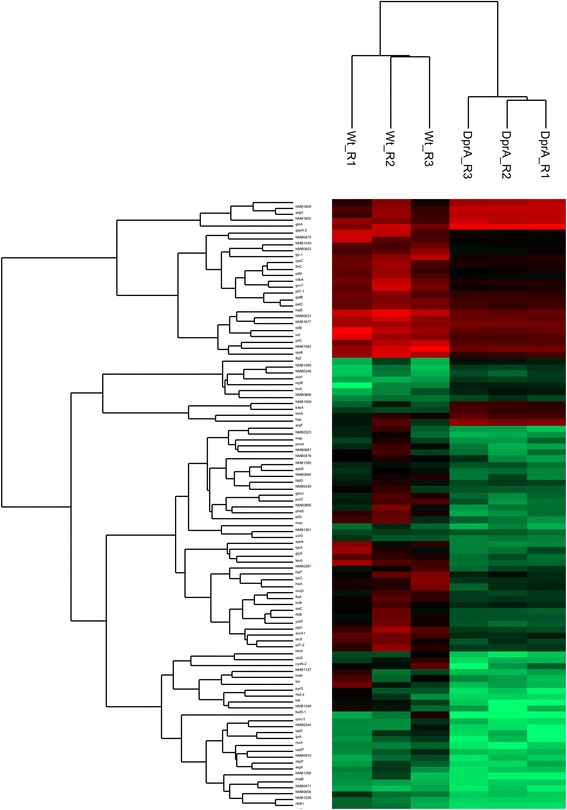
Unsupervised hierarchical clustering of LFQ values of 100 significantly abundant proteins (*p* ≤ 0.05, fold change =2) in *dprA* null mutant relative to the wildtype Nm. Samples with similar pattern of intensities of the significant proteins are clustered while dendrograms indicate cluster produced by Euclidean distance of proteins. Red and green colors indicate higher and lower abundances, respectively

For comparative analysis, protein groups with at least two valid LFQ values out of three biological replicates were considered. This criterion yielded a total of 1028 protein group eligible for quantitative analysis. Using a combination of *p ≤ 0.05* and a fold change cutoff of ±2, the analysis resulted in 100 proteins whose abundances were significantly changed (Fig. 2; Additional file 1: Table S1). Unsupervised hierarchical clustering (HC) of these significantly changed proteins showed that the LFQ values profile of *dprA* null mutant proteins clustered together distinctly from the wildtype protein samples (Fig. 3). Among the differentially abundant (DA) proteins, 16% were upregulated, and 84% were downregulated in the Nm *dprA* null mutant, respectively (Fig. 2; Additional file 1: Table S1).

### DprA affects proteins involved in recombination / 3R functions

**Fig. 4:**
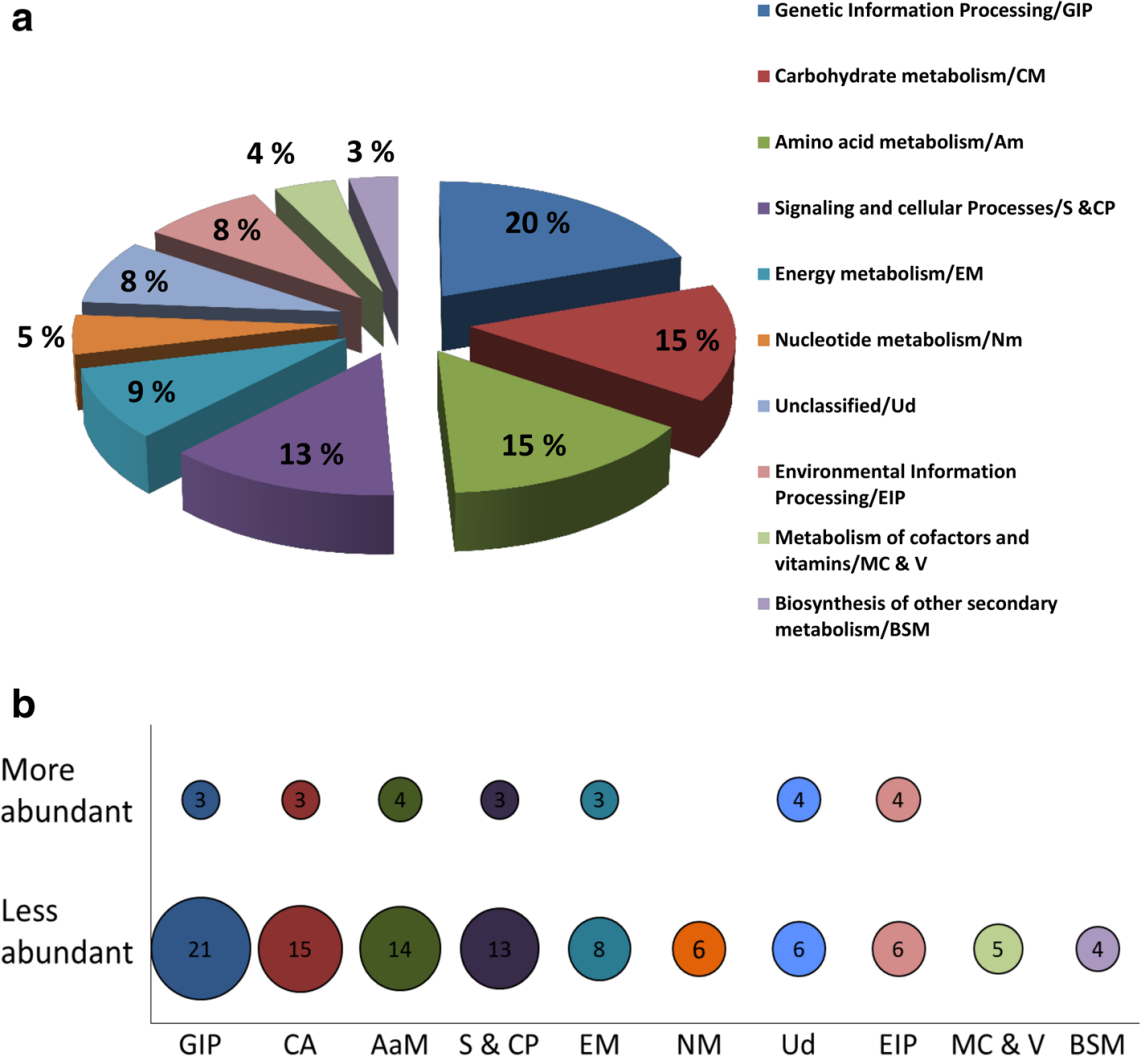
Function category of significantly differentially abundant (DA) proteins in Δ*dprA* mutant and the wild type Nm. A total of 100 DA protein sequences retrieved from UniProtKB were used as query dataset input and uploaded to BlastKOALA (KEGG Orthology and Links Annotation). The taxonomic group of genome, and the KEGG genes database file search used in the analysis were Prokaryotes:Bacteria, and species_prokaryotes.pep respectively. Approximately 92% of the DA proteins were functionally annotated (**a**). A bubble plot comparison of differentially less- and more- abundant proteins, the bubble size is proportional to the counts proteins (**b**). GIP - Genetic Information Processing; CM - Carbohydrate metabolism, AaM - Amino acid metabolism, S & CP - Signalling and cellular Processes, EM - Energy metabolism, NM - Nucleotide metabolism, Ud - Unclassified, EIP - Environmental Information Processing, MCV - Metabolism of cofactors and vitamins, and BSM - Biosynthesis of other secondary metabolism

The distribution of the Nm DA proteins enriched by the Kyoto Encyclopedia of Genes and Genomes (KEGG) category is shown in Fig. 4a and b. Most of the DA proteins belonged to the KEGG functional categories of genetic information processing, energy- and amino acid- metabolism, and signaling and cellular process. The genetic information processing proteins constitutes the DNA repair, recombination and replication (3R) proteins (Fig. 4). Notably, the RecA, UvrD, and HolA proteins (Table 1) which play roles in replication and homologous recombination were significantly downregulated in the Nm *dprA* null mutant relative to the wildtype. In addition, several of the 3R proteins including the replicative helicases RuvAB and DnaB, the DNA polymerase III (Pol III) β and ε subunits, DNA topoisomerase I, and DNA gyrase subunits A and B, as well as DNA translocase FtsK1 and FtsK2 were detected less abundant in the DprA mutant relative the wild type strain. However, the differences detected in the replication proteins were not significant. On the other hand, the single strand binding protein (SSB), DNA-binding protein (HupB), and the DNA Pol III γ and τ subunits were moderately more DA in the DprA mutant. This upregulation was not significant, yet detectable (Table 1).

**Table 1:** The 3R (replication, recombination and repair) gene products that were differentially abundant in the Nm Δ*dprA* mutant compared to the wildtype

Genes	Protein name/function	Protein fold change
*holA* ^a^	DNA polymerase III δ-subunit	−22.66
*recA* ^a^	Recombinase protein	−14.67
*ftsK2*	DNA translocase	−13.05
*recN*	DNA repair protein	−12.33
*gyrA*	DNA gyrase subunit A	−11.68
*mutS*	DNA mismatch repair protein	−11.59
*topA*	DNA topoisomerase 1	−10.19
*dnaQ-2*	DNA polymerase III ε-subunit	−7.30
*dnaB*	Replicative DNA helicase	−4.75
*dnaN*	DNA polymerase III β-subunit	−4.59
*rmuC*	DNA recombination protein RmuC homolog	−4.15
*ruvB*	ATP-dependent DNA helicase	−3.68
*gyrB*	DNA gyrase subunit B	−2.33
*uvrD* ^a^	DNA helicase	−1.78
*ftsK1*	DNA translocase	−1.60
*rdgC*	Recombination-associated protein	−1.52
*polA*	DNA polymerase I	−1.48
*ruvA*	ATP-dependent DNA helicase	−1.47
*dnaX*	DNA polymerase III subunits γ and τ	+2.07
*ssb*	Single-stranded DNA-binding protein	+2.37
*hupB*	DNA-binding protein HU-β	+4.27

^a^Significantly regulated proteins. The minus sign of the protein fold change indicates the downregulated whereas the positive sign indicates upregulated proteins

### DprA influences proteins involved in pilus biogenesis and twitching motility

A number of proteins involved in pilus biogenesis and twitching motility, the PilG, PilM, PilT1 and PilT2 (Table 2) were differentially less abundant in the Nm *dprA* null mutant relative to the wildtype strain. Notably, by MS analysis the amounts of PilG were 50X reduced in the DprA mutant. Conversely, DprA was 0.6X downregulated in the Nm Δ*pilG* mutant by MS analysis (Tables 2 and 3). Although not significantly regulated, the pilus assembly protein PilF, PilO, and the twitching motility protein (PilU, NMB0051) were found to be less abundant in the Nm DprA mutant, whereas PilE, PilQ, and the putative fimbrial biogenesis and twitching motility protein NMB1309 were slightly more abundant (Table 2).

**Table 2:** Gene products involved in transformation and pilus biogenesis that were differentially abundant in the Nm Δ*dprA* mutant compared to the wildtype when identified by mass spectrometry

Genes	Protein name	Protein fold change
*pilG* ^a^	Pilus assembly protein PilG	-50.33
*pilT-2* ^a^	Twitching motility protein PilT	-14.24
*pilT-1* ^a^	Twitching motility protein PilT	-11.75
*pilM* ^a^	PilM protein	-6.24
NMB0051 *(pilU)*	Twitching motility protein	-3.24
*pilF*	Type IV pilus assembly protein	-2.88
*pilO*	PilO protein	-2.57
*pilN*	PilN protein	-1.08
NMB0889 *(pilX)*	pilus assembly protein	-1.05
NMB0888 *(pilW)*	Putative type IV pilus assembly protein	+1.07
*pilP*	PilP protein	+1.39
*pilQ*	Type IV pilus biogenesis and competence protein PilQ	+1.50
NMB1309	Putative fimbrial biogenesis and twitching motility protein	+1.54
*pilE*	Fimbrial	+1.61

^a^Significantly downregulated proteins in the Δ*dprA* mutant. The minus sign of the protein fold change indicates the downregulated whereas the positive sign upregulated proteins

**Table 3:** LFQ values for DprA in *Neisseria meningitidis* wildtype and Δ*pilG* mutant detected by mass spectrometry

Nm strain	DprA LFQ1	DprA LFQ2	DprA LFQ3
Wildtype	3420300	1884100	2652200
NmΔ*pilG*	1244200	1823000	1533600

### Proteins involved in core metabolism are significantly regulated by DprA

The majority of proteins involved in cell division, carbohydrate- and energy-metabolism (e.g, the oxidoreductases), and amino acid metabolism were significantly regulated. For example, the cell division proteins FtsA (NMB0426), FtsZ (NMB0427), ZipA (NMB0667), MinD (NMB0171), and Lon (NMB1231) were downregulated when DprA was lacking. Also, the ATP-binding cassette (ABC transporters, NMB0387, NMB1240, and NMB1226) were significantly less abundant in the Nm *dprA* mutant, whereas the amino acid ABC transporter and the permease protein NMB1509 were significantly more abundant (Table 4). The Nm core metabolism components Pgm, CbbA, and TpiA of the glycolysis, NADH-quinone oxidoreductases NuoI (NMB0251), Mqo (NMB2096), Icd of the tricarboxylic acid (TCA) cycle, and the enzyme which catalyze the subpathway of leucine biosynthesis LeuA were downregulated in the lack of DprA. On the other hand some proteins involved in glycolysis (GapA-2), TCA cycle (NqrB), and amino acid metabolism (ArgF and ArgH) were upregulated in Nm *dprA* mutant (Table 4).

**Table 4:** Selected gene products significantly differentially abundant proteins in the Nm Δ*dprA* mutant compared to the wildtype when identified by mass spectrometry

Genes	Protein name/function	Protein fold change
Cell division proteins		
*ftsA*	Cell cycle	-7
*ZipA*	cell septum assembly	-24
*lon*	Lon protease/cell cycle	-22
*ftsZ*	Cell cycle	-12
*minD*	Septum site-determining protein	-11
Proteins of core metabolism		
Glycolysis and TCA cycle		
*tpiA*	Fructose and mannose metabolism; Glycolysis	-20.72
*pgm*	Amino sugar and nucleotide sugar metabolism	-19.52
*cbbA*	Fructose and mannose metabolism; Glycolysis	-10.60
*icd*	Carbon fixation; Citrate cycle (TCA cycle)	-8.59
*gapA-2*	Glyceraldehyde-3-phosphate dehydrogenase	5.70
*argH*	Arginine and proline metabolism	8.34
*argF*	Arginine and proline metabolism	12.90
Electron transport chain		
NMB1127	Oxidoreductase, short chain dehydrogenase	-56
*mqo*	Probable malate:quinone oxidoreductase	-27
*nuoI*	NADH-quinone oxidoreductase subunit I	-16
*nqrB*	Na(+)-translocating NADH-quinone reductase subunit B	+9
NMB0994	Acyl-CoA dehydrogenase family protein	-7
NMB0923	Cytochrome c	-6
NMB1677	Cytochrome c5	-5
*petC*	Ubiquinol cytochrome c reductase	-3
*kat*	Catalase	-22
*trxB*	Thioredoxin reductase	-8

The minus sign of the protein fold change indicates the less abundant whereas the positive sign denotes the more abundant proteins.

### Defense against oxidative stress is affected by DprA

Nm inhabits the oxygen-rich niche made up by the human oral mucosa and inevitably encounters continuous damage from the exposure to reactive oxygen species (ROS) [25]. Hence, multiple membrane proteins that are relevant in Nm pathogenesis and defense against oxidative stress proteins such as the Kat (Catalase), TrxB (thioredoxin reductase), and the c-type cytochromes [Cytochrome c (NMB0923), Cytochrome c5 (NMB1677), and the ubiquinol-cytochrome c reductase, cytochrome c1 (PetC)] were less abundant in the *dprA* null mutant (Table 4).

### DprA networks of proteins associated with the inner membrane

**Fig. 5:**
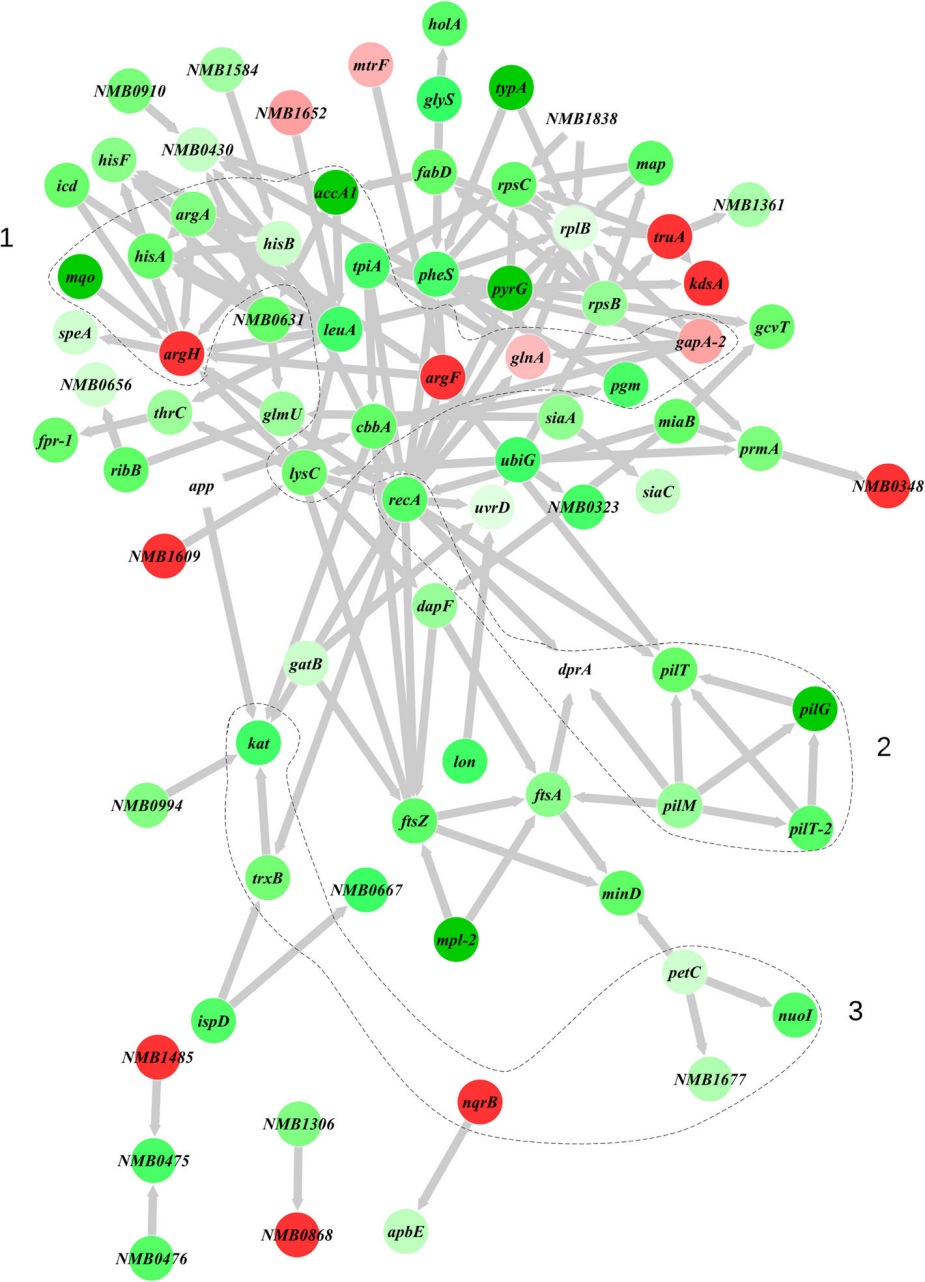
Network interaction analysis of proteins related to Nm. Known and predicted protein-protein interactions of the differentially abundant proteins were extracted from STRING database. Graphical representation of the interaction was generated by cytoscape software version 3.4. Proteins are represented as nodes, and the biological relationship between two nodes is represented as an edge (line). The intensity of the node color indicates the increased (red) or decreased (green) abundance according to fold changes. Those non-highlighted nodes were not in the input list but identified by STRING database as interacting partners. Dash-lined circle indicates sub-network of proteins linked to specific KEGG pathways. Core metabolic pathways (1); DNA transformation and recombination (2); Response to oxidative stress (3)

To define the functional and molecular interaction network, all the DA proteins were searched for in the online STRING protein query database. Eighty-one out of the 100 DA proteins exhibited evidence to be present in recognized and predicted networks with a total interaction edges of 147 (Fig. 5). This functional interaction network suggests that the RecA, PilT, FtsA, LeuA, ArgH, LysC and RpsB proteins represent the significant protein hubs. Interestingly, the three network sub-clusters of proteins (enclosed within the broken lines) were highly correlated to the following Nm functions: 1. DNA transformation, recombination, and pilus biogenesis, 2. core metabolism, and 3. response to oxidative stress (Fig. 5).

### DprA and PilG mutants exhibit mutual downregulation

**Fig. 6:**
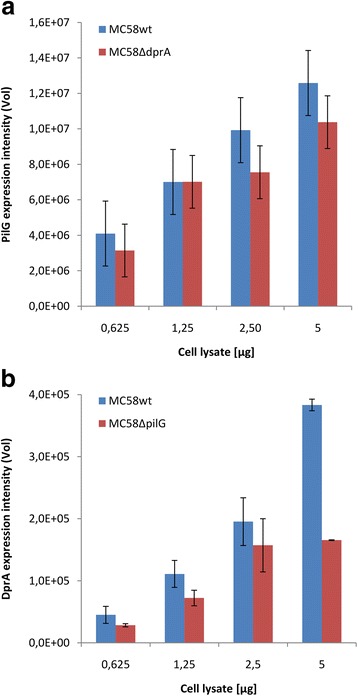
PilG and DprA immunoblots. MC58 wt and *ΔdprA* mutant overnight cultures were harvested in 1× PBS and heat inactivated at 60^0^C for 30 min. The cells were disrupted using MagNa Lyser (6× 90s at a speed of 6000 rpm). The cell lysates were prepared in Laemmli sample buffer and SDS-PAGE was run. The proteins transferred onto PVDF membrane. The membranes were incubated with ati-PilG (**a**) and ati-DprA (**b**) rabbit polyclonal primary antibodies, and anti-rabbit-IgG-horseradish peroxidase conjugate secondary antibody. The immunoblots were developed with Immun-Star WesternC Chemiluminescent kit (Bio-Rad) and visualized using a ChemiDoc Touch imager (Bio-Rad). Results were analysed using the Image Lab software (Bio-Rad)

By MS analysis, reduced amounts of PilG were detected in the *dprA* null mutant, and reduced amounts of DprA protein were detected in the Nm *pilG* null mutant. In order to validate the data obtained by MS analysis, we assayed the relative abundance of the DprA and PilG proteins by immunoblotting (Fig. 6, Additional file 2: Figure S2). The results from immunoblotting show that the amounts of these two proteins were mutually reduced in the null mutants. The level of PilG expression detected by immunoblotting was reduced in *dprA* null mutant Nm; however, the reduction of the level of expression was not significant. DprA levels were also reduced in the PilG null mutant Nm (Fig. 6). Further pull-down analysis by Co-IP coupled to MS analysis and immunoblotting revealed that most of the pilus biogenesis proteins that were detected, and/or identified less abundant in Δ*dprA* mutant in global proteome analysis, were found interact with DprA (Table 5, Additional file 3: Figure S3 A). The cell division proteins MinD, FtsZ, and FtsA which were significantly less abundant in the Δ*dprA* mutant, were found to interact with DprA. In addition, the recombinational repair proteins RecO, RecR, SSB, and TopA were detected as DprA-interacting partner proteins. Both PilG and PilE revealed interactions with PilT, PilN and SSB (Table 5). The detailed lists of the interacting partner proteins of DprA, PilG, or PilE are found in the Additional file 4 (Table S2). By MS analysis, the abundance of PilE in the Δ*dprA* mutant was shown to be equivalent to that of the abundance of PilE in the wildtype Nm, which was also confirmed by immunoblotting (Additional file 3: Figure S3 b, c). In the Δ*pilG* mutant, the lack of PilE and the presence of S-pilin were confirmed [26].

**Table 5:** List of proteins identified to interact with DprA or PilG by pull-down experiments with co-immunoprecipitation (Co-IP) and mass spectrometry (MS)

Protein IDs	Protein names	Gene names	Co-IP and MS identified proteins that interact with DprA, PilG, and/or PilE				
			Anti-DprA-Ab		Anti-PilG-Ab		Anti-PilE-Ab
			Wt	Δ*dprA*	Wt	Δ*pilG*	Wt
Q9K1K1	DNA processing chain A	*dprA*	**+**	**−**	**−**	**−**	**−**
Q7DDR1	Pilus assembly protein	*pilG*	**+**	**+**	**+**	**−**	**+**
P05431 Q4W588 Q4W587 Q4W586 Q4W589 Q4W585 Q4W590 Q4W592	Fimbrial protein; PilS cassette	*pilE & pilS*	**+**	**+**	**+**	**+**	**+**
Q4W563	PilN protein	*pilN*	**+**	**+**	**+**	**+**	**+**
Q70M91	Type IV pilus biogenesis and competence protein PilQ	*pilQ*	**+**	**+**	**−**	**+**	**−**
Q7DD78	PilO protein	*pilO*	**−**	**−**	**−**	**+**	**−**
Q9JY02	PilM protein	*pilM*	**−**	**+**	**−**	**−**	**−**
Q7DDU1	Twitching motility protein	*pilT-1*	**−**	**−**	**−**	**+**	**+**
P0A0S6	Cell division protein	*ftsZ*	**+**	**+**	**−**	**−**	**−**
Q9K0X8	Cell division protein	*ftsA*	**−**	**+**	**−**	**−**	**−**
Q7DDS7	Septum site-determining protein	*minD*	**+**	**+**	**−**	**−**	**−**
Q9JXD7	Probable malate:quinone oxidoreductase	*mqo*	**+**	**+**	**−**	**−**	**−**
Q7DDL4	Glutaredoxin	NMB0773	**−**	**−**	**−**	**+**	**−**
P0DH59	Protein RecA	*recA*	**−**	**+**	**+**	**+**	**−**
P66849	Single-stranded DNA-binding protein	*ssb*	**+**	**+**	**+**	**−**	**+**
Q9JZ92	Recombination protein	*recR*	**+**	**−**	**−**	**−**	**−**
Q9K0W0	DNA repair protein	*recO*	**+**	**−**	**−**	**−**	**−**
Q9K1K0	DNA topoisomerase 1	*topA*	**+**	**−**	**−**	**−**	**−**

Most of these proteins were also revealed to be significantly less abundant in the Nm Δ*dprA* mutant as compared to wildtype (wt) by MS of whole cell lysates. In Co-IP, DprA, PilG, or PilE was used as bait proteins through their reactivity with specific rabbit polyclonal antibodies (Ab). In order for DprA, PilG or PilE to target their interacting proteins, the Ab against the bait proteins were incubated with the cell lysates from Nm wt as well as the cell lysates from the Nm Δ*dprA* or Δ*pilG* mutant (in the mutant the bait protein is absent), and thereby the Ab binds the bait protein. The bait protein coupled with the antibody binds its interacting partner, forming the antibody-bait-prey protein complex. The “+” sign designates the formation of antibody-bait-prey protein complex whereas the “-” sign designates the absence of complex formation/interaction

## Discussion

DprA in bacteria is so far shown to be required for competence for DNA transformation [4] and suggested to be involved in RecA-mediated pilin variation [19]. The presence of a *dprA* gene has been suggested to be a distinctive feature of naturally transformable species [27]. DprA is ubiquitous in the microbial kingdom and its orthologs exist in certain eukaryotes, suggesting more fundamental functions for DprA. In one study, *dprA* was indicated to be one of the essential genes of Nm to cause invasive disease in an infant rat model [28]. However, beyond that, evidence of functions of DprA beyond transformation particularly its interaction with the pilus biogenesis and other proteins by Co-IP and proteome analysis has not formerly been presented. Here, we demonstrate that proteins involved in homologous recombination during transformation (RecA, UvrD and HolA), pilus biogenesis (PilG, PilM, PilT1, PilT2, PilE, PilQ, PilF, and PilO), and cell division, as well as core energy metabolism, and response to oxidative stress were less abundant in Nm *dprA* null cells (Fig. 5). Inverse downregulation of Nm DprA and PilG expression in the corresponding mutants indicate a link between DprA and the inner membrane protein PilG that could be attributed to either DNA processing or pilus biogenesis (Fig. 6). Notably, pull-down experiments using Co-Coupled to MS analysis demonstrated that DprA interacted with the pilus biogenesis protein PilG.

Genetic compensation where cells respond to the reduced expression of a gene by up-regulating compensatory genes/pathways is a well-known process [29, 30]. In this case, Nm responded to the loss of *dprA* expression by DA of a number of proteins involved in processes as diverse as recombinational-repair and replication, pilus biogenesis and twitching motility, cell division, energy-, and amino acid-metabolism (Fig. 4a and b).

In the *dprA* mutant, RecA was less abundant. RecA plays a central role in recombination and recombinational DNA repair [31]. The Holliday junction processing RuvAB [32], UvrD, and HolA were also downregulated proteins of the 3R genes. UvrD resolves Holliday junctions and is part of the mismatch and nucleotide excision repair pathways [33, 34]. HolA catalyzes DNA replication as a component of the Polymerase III holoenzyme [35]. However, in contrast to the reduced abundance of many of these 3R proteins, the survival of *dprA* mutant and the wild type Nm was virtually the same when exposed to genotoxic agents [36]. The survival of the *dprA* mutant equivalent to the wildtype Nm also contrasts with the downregulation of FtsK1 and FtsK2 proteins synchronizing chromosome segregation and cell division [37]. Furthermore, topoisomerases controlling the topology of DNA, TopA, GyrA, and GyrB [38, 39], were less abundant without DprA.

The pilus biogenesis proteins were among the less abundant differentially regulated proteins in Nm Δ*dprA* cells (Table 2; Fig. 6A). An interesting finding with regard to the function of DprA in transformation is that, components of the Tfp biogenesis proteins PilG, PilM, PilT1, and PilT2 proteins were less abundant in the Nm *dprA* mutant. DprA was also less abundant in the Nm Δ*pilG* mutant, suggesting a potential direct or indirect interaction between these components (Fig. 5). We have previously shown that PilG is absolutely required for Nm pilus biogenesis [26] and directly binds DNA [40, 41] and the secretin PilQ in transformation [40, 42]. The neisserial *pilG* and *pilT* mutants and the *Thermus thermophilus pilM* mutant are defective for transformation [43–46]. *T. thermophilus* PilM binds the inner membrane protein PilN, as well as ATP, and its structure is most similar to the actin-like protein FtsA [47]. In addition, PilT has been shown to be important in Nm pathogenesis and was upregulated in Nm grown in human blood [48]. The pilus subunit and biogenesis proteins essential for neisserial interaction with host cells and competence for transformation (PilE and PilC) [49, 50], including those required for DNA transformation PilQ, PilF, and PilO [42, 46, 51, 52], were found less abundant in the mutant strain. Nevertheless, the data presented here is not adequate to finally conclude that the synergistic effects of the reduced expression of proteins related to pilus biogenesis and RecA functions in the Nm DprA mutant strain have contributed for the transformation phenotype. Nm responds to the stress from growing in human blood by metabolic adaptation and energy conservation by downregulating some of the cell division genes, such as *minD* and *ftsZ* [53]. In our study, FtsA, FtsZ, ZipA, MinD, and Lon proteins were significantly less abundant in the lack of *dprA*. In addition, the direct interaction of Nm DprA with FtsZ and MinD in vitro was demonstrated. ZipA in association with FtsA facilitates the formation and stabilization of septum (FtsZ-ring) at midcell during cell division [54]. MinD is a membrane-associated ATPase which determines the site of septum formation [55]. Lon is a DNA dependant ATPase involved in cell division and production of capsular polysaccharide [56]. However, despite the reduced abundance of these proteins, the Nm *dprA* mutant exhibited growth phenotypes comparable to the wildtype strain [36]. One of the reasons for this observation could be that the levels of these cell division proteins were not a rate limiting factor in the growth conditions used, or because of other compensatory changes that occurred in the Nm *dprA* mutant [29, 30].

As a mucosal pathogen and survivor in the blood stream of humans, Nm needs to possess effective defense mechanisms to survive the oxidative stress produced by cells of the immune system [57]. In this context, the oxidoreductases important in the eukaryotes as well as in the bacterial respiratory chain and defense against oxidative stress [58] were less abundant in the Nm *dprA* mutant. For example, the short chain dehydrogenase/reductase (NMB1127) is a member of NAD(P)(H)-dependent oxidoreductases which play critical roles in lipid, amino acid, and carbohydrate metabolisms [59]. The NADH-quinone oxidoreductase NuoI (NMB0251) and Mqo (NMB2096) were also significantly downregulated. These oxidoreductases are primarily the main entry site for electrons to the respiratory chain of aerobic bacteria [60–62]. Particularly in Nm, the core metabolism was shown a key player in colonization and development of invasive diseases. In ex vivo transcriptomic analyses, genes encoding enzymes involved in glycolysis, such as Pgm and TpiA; Icd of the TCA cycle, and the majority of the NADH:ubiquinone oxidoreductase subunits were upregulated upon incubation of Nm cells in human blood [48, 53, 63]. *dprA* was also identified as one of the 73 genes required by Nm to develop septicemic disease in an infant rat model [28]. Electron transport chain-defective *Staphylococcus aureus* was reported to produce small colony variants highly sensitive to ROS produced by the host immune cells [62]. At the same time, proteins of the amino acid metabolism (ArgF and ArgH), glycolysis (GapA-2), and tricarboxylic acid (TCA) cycle (NqrB) were more abundant, which might account for compensatory mechanisms.

The c-type cytochromes are required in the final stage of the electron transport chain that is, they mediate the transfer of electrons from reduced cytochrome c to oxygen [64, 65]. Simultaneously, the c-type cytochromes contribute to the production of small amounts of superoxide anion and hydrogen peroxide [66]. The abundances of the c-type cytochromes (cytochrome c, cytochrome c5, and PetC) were all reduced in the lack of DprA. Nm MC58 mutants of the cytochromes c4 and putative cytochrome c (NMB0717) grow poorly under aerobic condition [64]. Previously, *petC* and cytochrome c5 (NMB1677) were shown to be upregulated in Nm grown in human blood [48, 53]. Nm cytochromes are important for growth under aerobic conditions, but also participate in the production of ROS [64, 66]. The enzymes catalyzing the detoxification of ROS, the catalase (Kat) and thioredoxin reductase (TrxB) were less abundant in *dprA* mutant. The Kat-including superoxide dismutases (SodB and SodC), glutathione peroxidase (GpxA), and disulfide oxidoreductase (DsbA) are among some of the ample Nm defenses against oxidative stress [67]. In Nm incubated with human blood cells, *kat* and *SodC* were upregulated [48]. TrxB was also reported to be important in the defense against oxidative- and disulfide-stress in *S. aureus* and *H. pylori* [58, 68]*.* In general, considering the absence of the membrane bound malate:quinone oxidoreductases in mammalian mitochondria whereas they are essential for growth [61] and the absence of DprA in mammals, implies that these proteins can be used as a potential drug target against pathogenic bacteria. Furthermore, the influence of the deletion of *dprA* on the core metabolism and the defense against oxidative stress indicates the importance of DprA for Nm pathogenesis.

The Nm ABC transporter protein NMB1240 was shown to be essential in causing septicemic disease in an infant rat model [28]. In the Nm *dprA* mutant, ABC transporter proteins NMB0387, NMB1240, and NMB1226 were significantly less abundant. ABC transporters are transmembrane permeases used for import and export of proteins, amino acids, sugars, ions or drugs in bacteria [69]. Besides, ABC transporters were found to be involved in competence for transformation, for example, the EcsB of *B. subtilis* [69] and the *adc* locus encoding an ABC transporter in *S. pneumoniae* [70]. Taken together, the results imply that DprA not only is directly involved in bacterial competence for natural transformation, but also acts indirectly with proteins from multiple cellular pathways.

Previous genetic and biochemical studies have shown that DprA is required for transformation; as for RMP, it was also shown that DprA interacts with SSB and the RecA [4, 8, 15–17, 20, 21]. The interaction and expression of some of the RMPs in neisserial transformation has previously been defined [71]. Proteins interact or are connected either by functioning in similar pathways or through direct or indirect regulatory networks.

In studies of genetic interactions, one of the customary approaches to understanding genetic interactions is to observe how individual proteins behave with respect to each other, either under certain environmental stress conditions, or under altered status of the cell which could be deletion of a gene [72, 73]. Proteomics is an important tool for the study of biological systems. The discovery of new diagnostic biomarkers and indirectly pursuing new therapeutic routes is broadly dependent on the discovery of significant dissimilarities/similarities between two cellular states to unravel the cellular and molecular mechanisms involved in a process; in this context, proteins are main targets as they carry out the major portion of cellular functions [74]. In an attempt to develop an effective vaccine against Nm serogroup B, comparative global proteomics of Nm serogroup B and the closely related but non-pathogenic *N. lactamica* was conducted to identify potential vaccine candidate proteins common to the two species [75]. Similar proteomic approaches were used in a pursuit for novel molecular therapeutic targets and potential Ng vaccine antigens [76, 77].

**Fig. 7:**
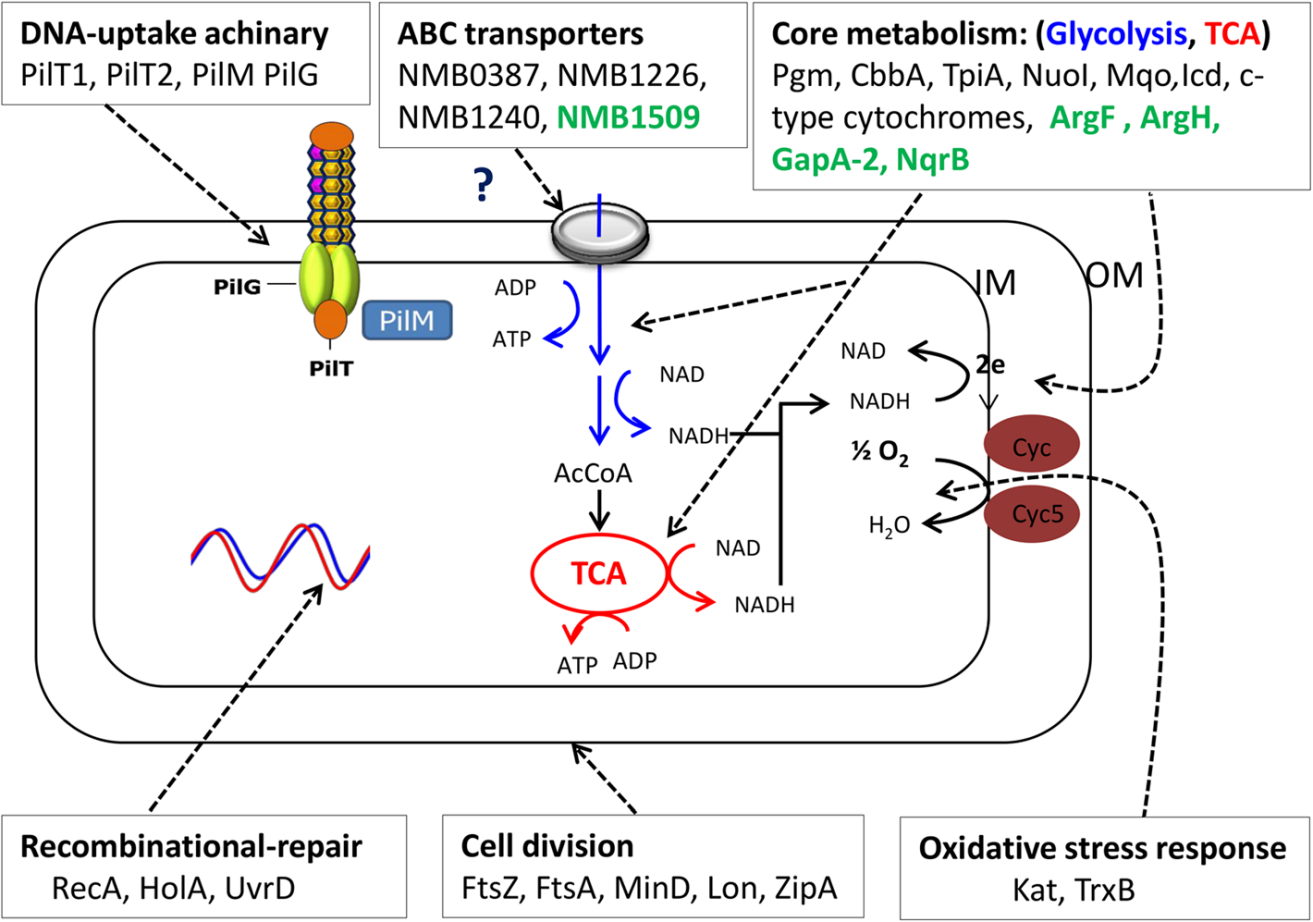
Schematic graphical presentation of the major pathway components or proteins significantly regulated in the *Neisseria meningitidis* (Nm)* dprA* mutant. Downregulated proteins are written in black text and upregulated in green. The figure illustrates: the limitation on the nutrient transport of Nm from the outside source possibly imposed by the reduced expression of ABC transporters and the subsequent effect on the Nm metabolism. The enzymes affected within the Nm core metabolism network (glycolysis, TCA cycle and electron transport chain). The less expressed enzymes involved in the production (cytochromes) and detoxification of ROS byproducts of metabolism. Components of the DNA-uptake machinery involved in the internalization of foreign DNA, proteins necessary for the integration of the internalized foreign DNA, DNA damage-repair, and replication, including the proteins required for proper division of the cell that are less expressed in the *dprA *mutant. IM- inner membrane, OM- outer membrane, Cyc- Cytochrome c, Cyc5- Cytochrome c5

Through pull-down experiments, MS and bioinformatics analyses, we demonstrated the global network of mainly inner membrane proteins and pathways affected in the lack of Nm DprA (Figs. 5 and 7)*.* Such an application of proteogenomics on bacterial wildtype and mutant cells represents a useful approach to enhance the output of traditional microbial genetics and bridging sub-disciplines of the -omics. Notably, the study suggests involvement of DprA directly or indirectly in Nm virulence together with the competence proteins important for pilus biogenesis and DNA transformation, as well as the recombinase RecA (Fig. 7). In the context of the already well known function of DprA, proteins involved in neisserial transformation through pilus biogenesis (PilG, PilM, PilT1, PilT2, PilE, PilQ, PilF, PilO, and the pilus substrate PilE) and recombination (RecA) were affected by the loss of DprA.

## Conclusion

We confirm that DprA is directly involved in competence for natural transformation via RecA loading and protection of the incoming ssDNA from degradation by restriction endonucleases [15, 78]. The depiction of DprA-related DA proteins, including the proteins that directly interact with DprA in a pull-down assay, also suggests an indirect role for DprA in colonization and pathogenesis by influencing the expression of Nm pilus biogenesis components as well as core inner membrane proteins that are important for adaptation to stress.

## Methods

### Strains and growth conditions

Nm strains serogroup B MC58 wildtype [79], the Δ*dprA* mutant (MC58 *dprA::aph)* [80] constructed by Hovland et al. [36], and the MC58 Δ*pilG* mutant [43] were grown on GC agar plates, or in CO_2_-saturated GC broth at 37 °C in 5% CO_2_. GC plates and broth were supplemented with 1% (*v*/v) IsoVitaleX. *E. coli* strains were grown in LB medium or on LB agar plates at 37 °C. When applicable, antibiotics were used in the following concentrations: 100 μg/ml ampicillin, 50 μg/ml kanamycin, 8 μg/ml erythromycin.

### Proteomic analysis

**i) Protein extraction.** Protein samples were prepared as depicted in Additional file 5: Figure S1. Overnight culture of Nm wild type and *ΔdprA* mutant cells were harvested into 15 ml tubes *(Sarstedt* ***,*** Germany***),*** washed three times in 1× PBS buffer, and heat inactivated at 60 °C for 30 min. The inactivated cell pellets were transferred into Lysing Matrix B tubes (Roche, US), resuspended in 2% SDS/10 mM Tris-HCl, pH 7.5 containing EDTA free protease inhibitor cocktail (Roche) and PhosStop (Roche) and mechanically disrupted by bead beating with MagNa Lyser (Roche, US) at speed for 90 s. The lysis procedure was repeated six times with 1 min cooling on ice in between. The lysate was clarified by centrifugation (15,000×*g* for 15 min) at 21 °C. The supernatant containing the lysate proteins were saved and concentration was determined by infrared spectrometry (DirectDetect™ Spectrometer, Millipore), aliquoted and stored at −80 °C until further use. **ii) Gel electrophoresis and in-gel digestion.** Proteins were separated based on their molecular weight by one-dimensional discontinuous sodium dodecyl sulfate polyacrylamide gel electrophoresis (SDS-PAGE) (1.0 mm, 4%–12% NuPAGE Novex Bis-Tris gel, Invitrogen). A working protein lysate of 100 μg was dissolved in 30 μl NuPAGE LDS sample buffer (4×) and NuPAGE sample reducing agent (10X) (Life Technologies). Samples were heated at 70 °C for 10 min before they were loaded onto 10-well NuPAGE gels. SDS-PAGE was run using 1× MOPS buffer at 80 V for 5 min followed by 20 min at 200 V. Gels were then stained using a colloidal Coomassie blue staining kit as per the manufacturer’s protocol. The entire protein gel lane was cut horizontally into six gel fractions. Gel slices were further cut into smaller pieces and destained at room temperature using 50% propanol in MQ (ultrapure) water, followed by dehydration in 100% propanol at 1000 rpm for 1 h.

After destaining, each fraction was subjected to in-gel reduction, alkylation, and trypsin digestion [81]. In brief, protein samples were reduced using 10 mM DTT for 1 h at 1000 rpm and 56 °C, and alkylated with 55 mM iodoacetamide (IAA) for 1 h at 1000 rpm and room temperature in dark. Gel fractions were further washed and dehydrated using 50% and 100% propanol, respectively, and vacuum-dried by Eppendorf 5301 concentrator. Reduced and alkylated samples were then saturated with 50 μl trypsin solution of 16.6 ng/μl sequence-grade porcine trypsin (Promega) in 50 mM ammonium bicarbonate. Gel fractions were then incubated at 4 °C for 2 h after which protein digestion was achieved by overnight incubation at 37 °C at 400 rpm [81].

The resulting peptide products were sequentially extracted using 100 μL of (50% and 100% acetonitrile) buffer at room temperature at 600 rpm for 20 min. The supernatant was transferred into new microtubes, concentrated in a vacuum-drier (Eppendorf 5301 concentrator) and resuspended in 30 μL of 0.05% (v/ v) trifluoroacetic acid (TFA). **iii) Co-immunoprecipitation (CO-IP) and** i**n-solution tryptic digestion:** to proteins that interact with DprA, PilG, and PilE, protein complexes were purified by Co-IP using Abs directed against DprA, PilG, or PilE as per the manufacturer’s protocol (Dynabeads Co-Immunopricipitation Kit, Novex life technologies) and analyzed by in-solution tryptic digestion MS. Briefly, the Co-IP purified protein complexes were lyophilized using a centrifugal vacuum concentrator without heat overnight, and solubilized in 30 μl urea buffer (6 M urea in 50 mM ammonium bicarbonate, ABC), reduced for 30 min at room temperature with 1 mM DTT and then alkylated for 15 min by 5 mM Iodoacetamide. The samples were diluted four fold in 50 mM ABC, digested by trypsin at a ratio of 1:100 *w*/w (trypsin: protein ratio) overnight at 37 °C with shaking at 400 rpm. Subsequently, the peptide products obtained both from in- gel and in-solution tryptic digest were desalted on reverse phase C_18_ Stop-and-Go Extraction (STAGE) tips [82]. The peptide samples were loaded onto ZipTip C_18_ tips activated and equilibrated with 95% ACN/0.1% FA and 0.1% formic acid (FA), respectively. The loaded peptide products were washed with 0.05% TFA and eluted with 95% ACN/0.1% FA. The eluent was dried using vacuum-drier (Eppendorf 5301 concentrator), dissolved in 0.1% formic acid (FA) and transferred to auto-sampler nano-LC vials prior to liquid chromatography tandem mass spectrometry (LC-MS/MS) analysis. **iv) Mass spectrometry analysis.** Peptide products were analyzed by nano-LC–MS/MS using a Q Exactive hybrid quadropole-orbitrap mass spectrometer interfaced with an EASY-spray ion source (Thermo Fisher Scientific) and coupled to a nano-LC HPLC (Easy nLC1000, Thermo). The peptides were loaded onto a trap column (C18, 100 μm × 2 cm, PepMap RSLC, Thermo Fisher Scientific) and separated on EASY-Spray columns (PepMapRSLC, C18, 2 μm particles, 100 Å, 50 cm, 75 μm ID, Thermo Scientific) using a 2 h binary gradient as follow: from 2% to 30% solvent B in 90 min followed by 30% to 45% solvent B in100–115 min and kept at 90% B until 120 min at a flow rate of 0.3 μL/min. Solvents used were 0.1% FA/3% acetonitrile (solvent A) and 0.1% FA in 100% ACN as solvent B). The column was operated at constant temperature of 60 °C. The LC was coupled to a Q Exactive mass spectrometer via an Easy nanoelectrospray source (Thermo Scientific). The MS instrument was operated in data dependent acquisition mode with automatic switching between MS and MS/MS scans. Full MS scans were acquired in resolution of 70,000, with automatic gain control target value of 3 × 10^6^ ions or maximum injection time of 100 ms within the scan range 400–1200 m/z. Peptide fragmentation was performed by higher energy collision dissociation (HCD) with normalized collision energy set to 25. The MS/MS spectra were acquired of the 10 most abundant ions (top 10 method) in the resolution *R* = 17,500, automatic gain control target value of 1 × 105 ions, or maximum fragment accumulation time of 200 ms. An isolation window of 3 Da was used. **v) Database searching and analysis.** Analysis of MS data was performed using MaxQuant software package (version 1.4.0.5) as described by J Cox and M Mann [83]. Tandem mass spectra (MS/MS) were searched by the Andromeda search engine [84] against the UniProtKB FASTA database for the *Nm* serogroup B (strain MC58) (2001 entries, downloaded from www.UniProt.org April 2016) using the following parameters: Enzyme specificity was set as Trypsin/P, and a maximum of two missed cleavages and a mass tolerance of 0.5 Da for fragment ion were applied. The ‘requantify’ and ‘match between runs’ options were checked with a retention time alignment window of 3 min. Oxidations (M), acetylation (protein N term), Gln-pyro (Q) and pyro-Glu (E) were specified as variable modifications and carbamidomethyl (C) as fixed modification.

Database searches were performed with a mass tolerance of 20 ppm for precursor ion for mass calibration, and with a 6 ppm tolerance after calibration. The maximum false peptide and protein discovery rate was specified as 0.01. Seven amino acids were required as minimum peptide length. Proteins with at least two peptides of which at least one is unique were considered as reliably identified. Following protein identification by a database search, validation for multiple comparisons was corrected using the Benjamini-Hochberg correction [85]. To aid in the control of false positives, the database was supplemented with additional sequences for common contaminants and reversed sequence of each entry. The default settings were applied for all other parameters. **vi) Statistics and ontology analysis.** The statistical determination of protein abundances was assessed using Perseus software (version 1.5.1.6, http://www.perseus-framework.org) as previously described [86]. We used the proteins groups output from MaxQuant as the basis for all the subsequent statistical and ontology enrichment analysis. Intensity based absolute quantification (LFQ) values were used to assess differences in the abundance of proteins between the three biological replicates of Nm wildtype and *ΔdprA* mutant cells. Briefly, the protein groups output was filtered by removing matches to the reverse database, matches only identified by site, and common contaminants. Subsequently, LFQ values were transformed to log_2_. For quantitation, at least two valid LFQ values out of the three biological experiments were required. Signals that were originally zero (missing values) were imputed with random numbers from a normal distribution, whose mean and standard deviation were chosen to best simulate low abundance values below the noise level (width = 0.3; shift =1.8) [86].

To identify proteins whose abundance were significantly differentially abundant between the wildtype and *ΔdprA* mutant, a two tailed unpaired t test was used using a false discovery rate (FDR) value of 0.05 and S0 = 2. The S0 parameter sets a threshold for minimum fold change [87]. The resulting significant proteins were analyzed for annotation enrichments. A two-tailed Fisher’s exact test was used to assess the significance of enrichment terms. Proteins assigned to enriched term categories (*p*-value < 0.05) were grouped according to the KEGG classification. **Vii) Proteins interaction network analysis.** To further interpret the biological significance of differentially regulated proteins in terms of protein-protein interaction networks, we used the search tool for the retrieval of interacting genes version 10.0 [88] (STRING, http://string-db.org/) online tool. The required minimum interaction score of at least 0.4 was used as the cut-off criterion. Then, Cytoscape software [89] was used to visualize the interaction network. The properties of the network including node degree and edge attributes were analyzed. Nodes represent proteins and edges represent the interactions/connections between proteins. The degree represents the number of interactions associated with the protein. Proteins with a large degree are known as hub proteins [90] and they are considered to be the essential or key protein in the network [91]. The Network Analyzer option in Cytoscape 3.4.1 was used to compute the degree and betweenness centrality of the network [92].

### Immunoblotting

Whole cell lysates from MC58 were separated by SDS-PAGE (Novex), and transferred onto polyvinylidene fluoride (PVDF) membrane. Membranes were washed with Tris buffered saline (TBS) buffer containing 0.05% (*v*/v) Tween 20. Blocking was performed with non-fat dry milk. Primary antibody incubation was overnight at 4°C with affinity purified rabbit polyclonal antibodies raised against recombinant protein. Secondary antibody incubation with anti-rabbit-IgG-horseradish peroxidase conjugate was performed at room temprature for 4 h. The immunoblots were developed using the Immun-Star WesternC Chemiluminescent kit (Bio-Rad) and visualized using a ChemiDoc Touch imager (Bio-Rad). Results were analysed using the Image Lab software (Bio-Rad).

### Co-immunoprecipitation-coupled MS and immunoblotting

Pull-down experiments by Co-IP and MS analysis was used to further investigate DprA, PilG, and PilE interactions to each other and with other proteins by incubating the cell lysates with polyclonal rabbit Abs raised against DprA and PilG recombinant proteins and purified Tfp (PilE). For this purpose, Nm strains MC58 wildtype, Δ*dprA,* and Δ*pilG* were grown overnight (ON) on GC agar plates. The next day, piliated colonies were picked under a stereo-microscope, and uniformly cultured on GC agar plates. From the ON culture, between 1 and 1.2 g of cells were harvested and washed three times in 1% phosphate-buffered saline, pH 7.4 (PBS). The cell pellets were lysed by the detergent lysis method according to the manufacturer’s protocol (Dynabeads Co-Immunoprecipitation Kit, Novex life technologies) with minor modifications. Briefly, the cell pellets were resuspend in 1:9 ratio of cell mass to extraction buffer [100 mM NaCl, 2 mM MgCl, 1 mM DTT, and 1% n-Dodecyl β-D-maltoside (DDM)] containing protease inhibitors, and incubated on ice for 15 min. The cell lysates were centrifuged at 5000×g for 5 min at 4 °C, and the supernatants were immediately used for co-immunoprecipitation. Appropriate amount of Dynabeads coupled to anti-DprA or anti-PilG antibodies according to the manufacturer’s protocol were added to the cell lysates, and incubated on a rotator at 4 °C for 1.5 h. The incubation allowed DprA or PilG proteins to bind their respective antibody-coupled Dynabeads; in turn, DprA or PilG bound their interacting protein partners within the cell lysate, yielding protein complexes. Finally, the Dynabeads were washed, and the protein complexes were eluted by the elution buffer supplied with the kit. The eluted protein complex samples were analyzed by MS (as described in proteomic analysis sections iii-v), and immunoblotting as described above, except that the immunoblots were detected using 5-bromo-4-chloro-39-indolyl phosphate p-toluidine salt (BCIP) and nitro-blue tetrazolium chloride (NBT) as substrates for the AP [41].

## Additional files

Additional file 1: Table S1. Complete list of all the significantly differentially abundant proteins identified by mass spectrometry. (PDF 152 kb)

Additional file 2: Figure S2. PilG and DprA expression is reduced in Nm Δ*dprA* and Δ*pilG* mutants, respectively. A) Representative gel images of the Δ*dprA* mutant-*,* and B) Δ*pilG* mutant-, compared to the wild type Nm as analysed by western blot. In both A and B lanes 1–4 represent 0.625, 1.25, 2.5 and 5 μg of cell lysates from Nm wild type, Lane 5 in A and B are negative controls that is, cell lysates from Δ*pilG* and Δ*dprA,* respectively*.* In both A and B lane 6–9 contains 0.625, 1.25, 2.5 and 5 μg of cell lysates from the corresponding Nm knockout mutant strains. (TIFF 931 kb)

Additional file 3: Figure S3. DprA interacts with PilG. A) PilG co-immunoprecipitated using anti-DprA antibody from the cell lysates, and confirmed by western blot using anti-PilG antibody. Cell lysates samples indicated by lane 1, 2, and 3 were from the Nm MC58 wild type, Δ*dprA*, and Δ*pilG,* respectively). Also, the presence of PilG band at lane 2 suggests the direct interaction and co-immunoprecipitation of anti-DprA antibody with PilG in the absence of DprA. B) Western blot by anti-PilE antibody showing that PilE expression of the Nm Δ*dprA* mutant is comparable with the wild type Nm PilE expression; 20 and 40 μg cell lysates were used from each sample; lanes 1–2, 3–4, and 5–6 indicate lysates from Nm wild type, Δ*dprA*, and Δ*pilG*, respectively. The band corresponding to PilE is absent in Δ*pilG* mutant Nm, lane 5 and 6. C) Single point quantitation of PilE western blot using Image Studio Lite analysis software. (TIFF 759 kb)

Additional file 4: Table S2. Proteins identified interact with DprA, PilG, or PilE by Co-Immunopricipitation (Co-IP). During CO-IP DprA, PilG, or PilE was used as bait proteins. In order for DprA, PilG or PilE target their interacting proteins, the antibody (Ab) against the bait proteins were incubated with the cell lysates from Nm wild type (Wt), also incubated with the cell lysates from Δ*dprA,* or Δ*pilG* mutant Nm (in the mutant the bait protein is absent), subsequently the antibody bind the bait protein. The bait protein coupled with the antibody binds its interacting partner, and form antibody-bait-prey protein complex. The “+” sign designates the formation of antibody-bait-prey protein complex whereas the “-” sign designates the absence of complex formation/interaction. (PDF 256 kb)

Additional file 5: Figure S1. Schematic diagram of the proteomics work flow. MC58 wt and Δ*dprA* mutant overnight (ON) cultures were harvested, washed 3× with 1× PBS, centrifuged at 6000 RPM for 10 min. The pellets were resuspend in lysis buffer, transferred into Lysing Matrix B tubes containing 0.1 mm silica beads, and disrupted using MagNa Lyser (6× 90s at a speed of 6000). The cell lysates cleared by spinning down at 15,000×g for 15 min, and the supernatant (containing protein) were analyzed by SDS-PAGE. The gel lanes were cut into six pieces, and digested with trypsin. The peptides products were extracted and purified, and injected into an electrospray-based Q-Exactive MS. The MS output proteins were identified and quantified using MaxQuant as described in method section. Finally, the differentially expressed proteins were functional annotated (KEGG). (TIFF 2976 kb)

## Abbreviations

ABC: ATP-binding cassette
Co-IP: co-immunoprecipitation
DA: Differentially abundant
DprA: DNA processing chain A
KEGG: Kyoto Encyclopedia of Genes and Genomes
LFQ: Label free quantitative
MS: Mass spectrometry
NAD: Nicotinamide adenine dinucleotide
Nm: *Neisseria meningitidis*
ROS: reactive oxygen species
SSB: single-strand binding protein
TCA: the tricarboxylic acid cycle
