# Supplementary material for: Comparative proteomic analysis of Neisseria meningitidis wildtype and dprA null mutant strains links DNA processing to pilus biogenesis
Source: BMC Microbiol. 2017 Apr 21;17:96. doi: 10.1186/s12866-017-1004-8 (PMC5399837; doi:10.1186/s12866-017-1004-8)
Supplement: Supplementary file 1 — Complete list of all the significantly differentially abundant proteins identified by mass spectrometry. (PDF 152 kb) [file 12866_2017_1004_MOESM1_ESM.pdf]

Table S1. All the significantly differentially expressed proteins in *Neisseria meningitidis dprA* mutant strain relative to the wildtype as identified by mass spectrometry. Protein fold changes are log2-transformed t-test difference values. The minus sign indicate downregulated whereas the positive numbers show upregulated proteins.

Gene	Protein name	Protein IDs	Fold change
NMB0387	ABC transporter, ATP-binding protein	Q9K112	-71,311
NMB1348	Putative RNA methylase	Q9JZ06	-67,597
NMB1127	Oxidoreductase, short chain dehydrogenase/reductase family	Q9JRT0	-56,265
pilG	Pilus assembly protein PilG	Q7DDR1	-50,334
typA	GTP-binding protein TypA		-47,747
pyrG	CTP synthase	Q9JYJ8	-44,748
rpmJ1	50S ribosomal protein L36 1	P66293	-41,877
cysN-2	Sulfate adenylyltransferase subunit 1	Q9JRU7	-38,504
fadD-1	Long-chain-fatty-acid--CoA ligase	Q9JZ62	-29,641
accA1	Acetyl-coenzyme A carboxylase carboxyl transferase subunit alpha	Q9JRV8	-28,933
mpl-2	UDP-N-acetylmuramate:L-alanyl-gamma-D-glutamyl-meso-diaminopimelate ligase	Q9JRY9	-28,749
mqo	Probable malate:quinone oxidoreductase	Q9JXD7	-27,268
glyS	Glycine--tRNA ligase beta subunit	Q9JXQ5	-26,415
NMB0667	Cell division protein ZipA	Q9K0E2	-23,805
ubiG	Ubiquinone biosynthesis O-methyltransferase	Q9JXI7	-23,447
NMB0895	UPF0246 protein NMB0895	Q9JZU5	-23,435
leuA	2-isopropylmalate synthase	Q9JZG1	-22,644
lon	Lon protease	Q9JZ95	-22,411
kat	Catalase	Q7DDS5	-22,219
pheS	Phenylalanine--tRNA ligase alpha subunit	Q9K092	-21,018
tpiA	Triosephosphate isomerase	Q9JXT8	-20,717
NMB0323	UbiH family protein	Q9K157	-20,294
pgm	Phosphoglucomutase	P40391	-19,515
NMB0475	Uncharacterized protein	Q9K0T7	-19,233
purC	Phosphoribosylaminoimidazole-succinocarboxamide synthase	Q9K063	-18,202
ispD	2-C-methyl-D-erythritol 4-phosphate cytidylyltransferase	Q9JYM4	-16,091
nuoI	NADH-quinone oxidoreductase subunit I	Q7DDS1	-15,593
recA	Protein RecA	P0DH59	-14,674
NMB0476	Uncharacterized protein	Q7DDQ0	-14,642
holA	DNA polymerase III, delta subunit	Q9K0A6	-14,296
pilT-2	Twitching motility protein PilT	Q9K053	-14,241
iscS	Cysteine desulfurase IscS	Q9JYY0	-12,974
NMB1395	Alcohol dehydrogenase, zinc-containing	Q9JYX0	-12,260

Gene	Protein name	Protein IDs	Fold change
ftsZ	Cell division protein FtsZ	P0A0S6	-12,251
map	Methionine aminopeptidase	Q9JXD9	-12,186
pilT-1	Twitching motility protein PilT	Q7DDU1	-11,754
ribB	3,4-dihydroxy-2-butanone 4-phosphate synthase	Q9JZ77	-11,645
minD	Septum site-determining protein MinD	Q7DDS7	-11,044
NMB1240	ABC transporter, ATP-binding protein	Q9JZ89	-10,633
cbbA	Fructose-bisphosphate aldolase	Q9JXV5	-10,600
ychF	Ribosome-binding ATPase Y	Q7DD71	-10,567
rpsC	30S ribosomal protein S3	P66551	-10,558
NMB0631	Putative phosphate acetyltransferase Pta	Q9K0H1	-9,956
hisA	1-(5-phosphoribosyl)-5-[(5-phosphoribosylamino)methylideneamino] imidazole-4-carboxamide isomerase	Q9K0H3	-9,623
fabD	Malonyl CoA-acyl carrier protein transacylase	Q9JXR4	-9,507
miaB	tRNA-2-methylthio-N(6)-dimethylallyladenosine synthase	Q9JXV8	-9,382
uppP	Undecaprenyl-diphosphatase	Q9K0Z3	-9,221
fpr-1	Ferredoxin--NADP reductase	Q7DDI7	-8,868
gcvT	Aminomethyltransferase	Q9K0L8	-8,864
icd	Isocitrate dehydrogenase, NADP-dependent, monomeric type	Q9JZS1	-8,816
lysC	Aspartokinase	Q9JYN6	-8,592
NMB1226	ABC transporter, ATP-binding protein	Q9JZ98	-8,461
trxB	Thioredoxin reductase	Q9JZ28	-8,269
NMB0910	Transcriptional regulator	Q9JZT1	-7,943
prmA	Ribosomal protein L11 methyltransferase	Q9JXW2	-7,749
NMB0344	BolA/YrbA family protein	Q9K145	-7,572
argA	Amino-acid acetyltransferase	Q9JXU9	-7,568
NMB1306	Uncharacterized protein	Q7DDC7	-7,447
NMB0994	Acyl-CoA dehydrogenase family protein	Q9JZL9	-7,397
hisF	Imidazole glycerol phosphate synthase subunit HisF	Q9K0H4	-7,014
prlC	Oligopeptidase A	Q9K1E2	-7,003
ftsA	Cell division protein FtsA	Q9K0X8	-6,942
siaA	UDP-N-acetylglucosamine 2-epimerase	H2VFI5	-6,639
rpsB	30S ribosomal protein S2	P66540	-6,399
pilM	PilM protein	Q9JY02	-6,235
dapF	Diaminopimelate epimerase	Q9K060	-6,132
glmU	Bifunctional protein GlmU	Q9K1P3	-5,914
thrC	Threonine synthase	Q9JZH7	-5,913
NMB0923	Cytochrome c	Q9JZR9	-5,620
NMB1584	3-hydroxyacid dehydrogenase	Q9JYH6	-5,578
NMB1361	Pseudouridine synthase	Q9JYZ4	-5,057
rfbB1	dTDP-glucose 4,6-dehydratase	P55294	-5,010

Gene	Protein name	Protein IDs	Fold change
NMB1677	Cytochrome c5	Q9JYA2	-4,745
apbE	Thiamine biosynthesis lipoprotein ApbE	Q9K0M9	-3,894
siaC	Polysialic acid capsule biosynthesis protein SiaC	Q7DDU0	-3,597
NMB0430	Methylisocitrate lyase	Q9K0X5	-3,400
hisB	Imidazoleglycerol-phosphate dehydratase	P64371	-3,174
gatB	Aspartyl/glutamyl-tRNA(Asn/Gln) amidotransferase subunit B	Q9JYZ7	-3,123
petC	Ubiquinol-cytochrome c reductase, cytochrome c1	Q9JXH1	-2,947
speA	Biosynthetic arginine decarboxylase	Q9K0U3	-2,823
NMB0471	Uncharacterized protein	Q9K0U1	-2,785
NMB0656	Uncharacterized protein	Q9K0F1	-2,735
rplB	50S ribosomal protein L2	Q9K1I5	-1,876
uvrD	DNA helicase	Q9K1D0	-1,778
NMB1509	Amino acid ABC transporter, permease protein	Q7DDB4	3,188
hap	Adhesion and penetration protein	Q9JXL6	3,404
glnA	Glutamine synthetase	Q9K134	5,052
mtrF	Efflux pump component MtrF	Q9JY63	5,701
gapA-2	Glyceraldehyde-3-phosphate dehydrogenase	Q9JX95	6,921
NMB1652	UPF0210 protein NMB1652	Q9JYC3	7,104
NMB0868	Uncharacterized protein	Q9JZW7	8,095
argH	Argininosuccinate lyase	Q9K0G8	8,344
NMB1609	Trans-sulfuration enzyme family protein	Q9JYF3	8,441
nqrB	Na(+)-translocating NADH-quinone reductase subunit B	Q9K0M4	8,973
NMB0348	tRNA-dihydrouridine synthase	Q9K142	9,436
NMB1485	Uncharacterized protein	Q9JYP7	10,530
trmA	tRNA/tmRNA (uracil-C(5))-methyltransferase	Q9JYA0	11,972
kdsA	2-dehydro-3-deoxyphosphooctonate aldolase	Q9JZ55	12,883
argF	Ornithine carbamoyltransferase	P0DH57	12,907
truA	tRNA pseudouridine synthase A	Q9JXI2	18,331
