# Supplementary figures and images for: Comparative proteomic analysis of Neisseria meningitidis wildtype and dprA null mutant strains links DNA processing to pilus biogenesis
Source: BMC Microbiol. 2017 Apr 21;17:96. doi: 10.1186/s12866-017-1004-8 (PMC5399837; doi:10.1186/s12866-017-1004-8)

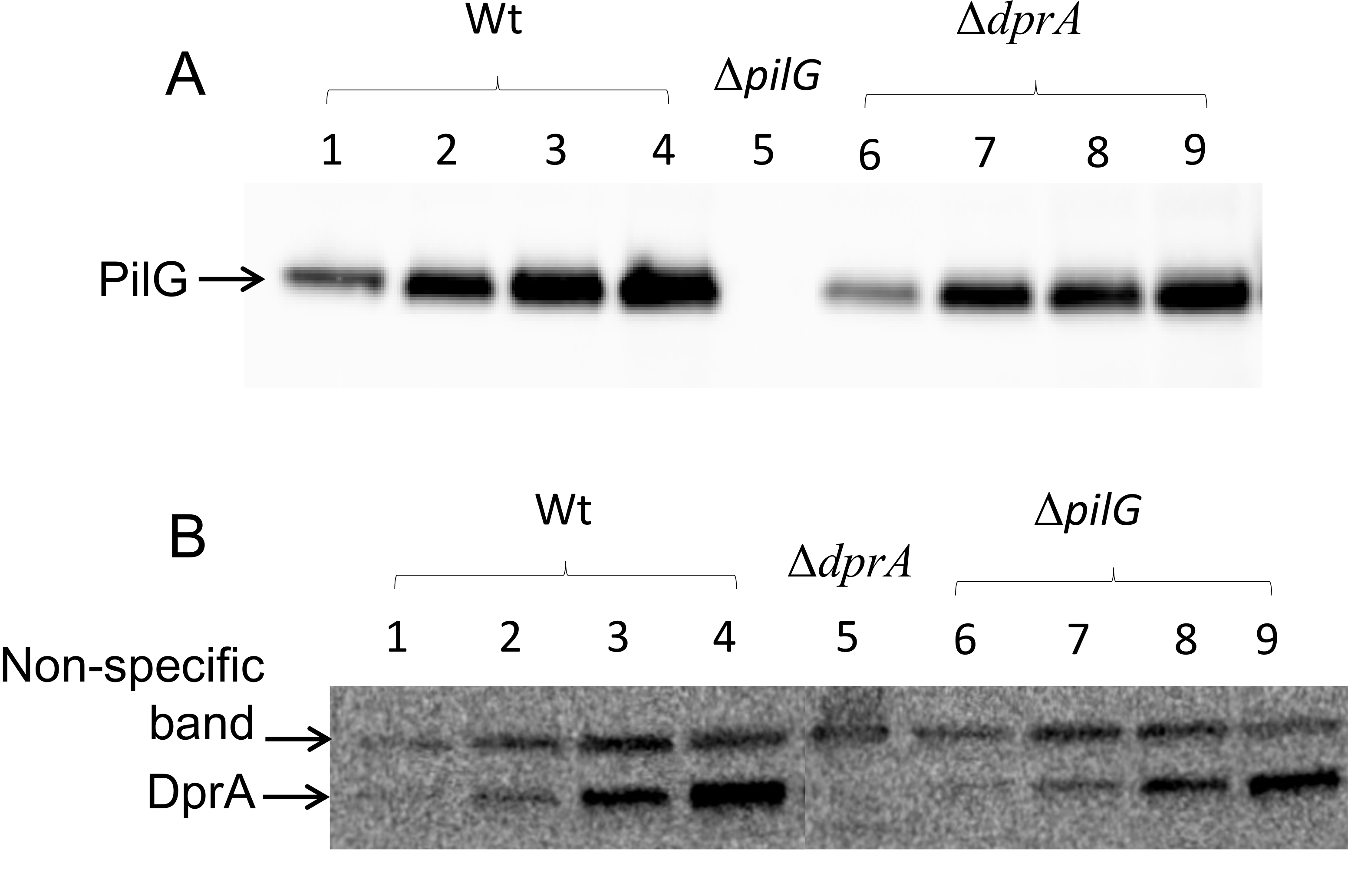

Supplement: Supplementary file 2 — PilG and DprA expression is reduced in Nm ΔdprA and ΔpilG mutants, respectively. A) Representative gel images of the ΔdprA mutant-, and B) ΔpilG mutant-, compared to the wild type Nm as analysed by western blot. In both A and B lanes 1–4 represent 0.625, 1.25, 2.5 and 5 μg of cell lysates from Nm wild type, Lane 5 in A and B are negative controls that is, cell lysates from ΔpilG and ΔdprA, respectively. In both A and B lane 6–9 contains 0.625, 1.25, 2.5 and 5 μg of cell lysates from the corresponding Nm knockout mutant strains. (TIFF 931 kb) [file 12866_2017_1004_MOESM2_ESM.tif]

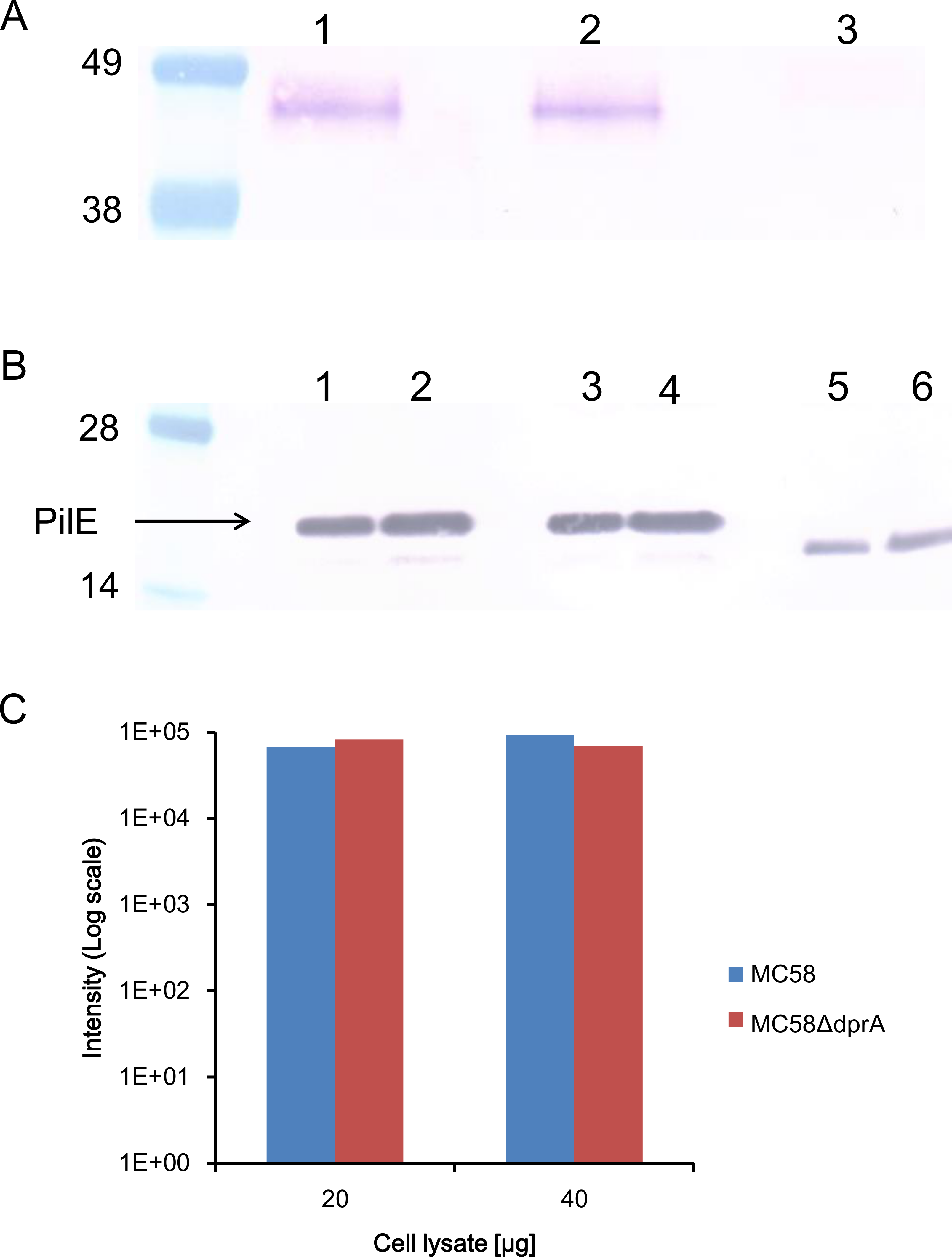

Supplement: Supplementary file 3 — DprA interacts with PilG. A) PilG co-immunoprecipitated using anti-DprA antibody from the cell lysates, and confirmed by western blot using anti-PilG antibody. Cell lysates samples indicated by lane 1, 2, and 3 were from the Nm MC58 wild type, ΔdprA, and ΔpilG, respectively). Also, the presence of PilG band at lane 2 suggests the direct interaction and co-immunoprecipitation of anti-DprA antibody with PilG in the absence of DprA. B) Western blot by anti-PilE antibody showing that PilE expression of the Nm ΔdprA mutant is comparable with the wild type Nm PilE expression; 20 and 40 μg cell lysates were used from each sample; lanes 1–2, 3–4, and 5–6 indicate lysates from Nm wild type, ΔdprA, and ΔpilG, respectively. The band corresponding to PilE is absent in ΔpilG mutant Nm, lane 5 and 6. C) Single point quantitation of PilE western blot using Image Studio Lite analysis software. (TIFF 759 kb) [file 12866_2017_1004_MOESM3_ESM.tif]

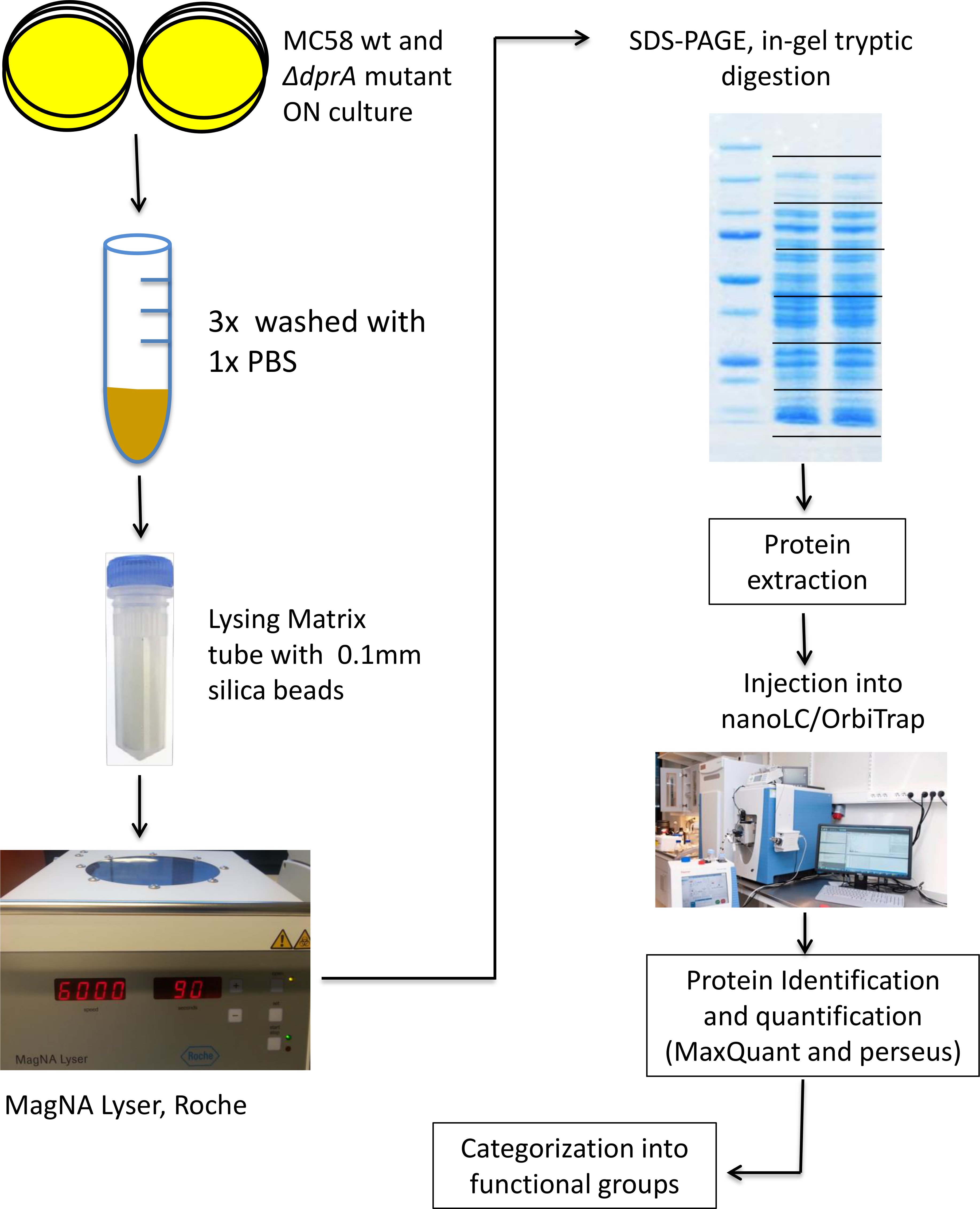

Supplement: Supplementary file 5 — Schematic diagram of the proteomics work flow. MC58 wt and ΔdprA mutant overnight (ON) cultures were harvested, washed 3× with 1× PBS, centrifuged at 6000 RPM for 10 min. The pellets were resuspend in lysis buffer, transferred into Lysing Matrix B tubes containing 0.1 mm silica beads, and disrupted using MagNa Lyser (6× 90s at a speed of 6000). The cell lysates cleared by spinning down at 15,000×g for 15 min, and the supernatant (containing protein) were analyzed by SDS-PAGE. The gel lanes were cut into six pieces, and digested with trypsin. The peptides products were extracted and purified, and injected into an electrospray-based Q-Exactive MS. The MS output proteins were identified and quantified using MaxQuant as described in method section. Finally, the differentially expressed proteins were functional annotated (KEGG). (TIFF 2976 kb) [file 12866_2017_1004_MOESM5_ESM.tif]
