# Supplementary material for: Comparative proteomic analysis of Neisseria meningitidis wildtype and dprA null mutant strains links DNA processing to pilus biogenesis
Source: BMC Microbiol. 2017 Apr 21;17:96. doi: 10.1186/s12866-017-1004-8 (PMC5399837; doi:10.1186/s12866-017-1004-8)
Supplement: Supplementary file 4 — Proteins identified interact with DprA, PilG, or PilE by Co-Immunopricipitation (Co-IP). During CO-IP DprA, PilG, or PilE was used as bait proteins. In order for DprA, PilG or PilE target their interacting proteins, the antibody (Ab) against the bait proteins were incubated with the cell lysates from Nm wild type (Wt), also incubated with the cell lysates from ΔdprA, or ΔpilG mutant Nm (in the mutant the bait protein is absent), subsequently the antibody bind the bait protein. The bait protein coupled with the antibody binds its interacting partner, and form antibody-bait-prey protein complex. The “+” sign designates the formation of antibody-bait-prey protein complex whereas the “-” sign designates the absence of complex formation/interaction. (PDF 256 kb) [file 12866_2017_1004_MOESM4_ESM.pdf]

Table S2. Proteins identified to interact with DprA, PilG, or PilE by co-immunoprecipitation (Co-IP) and mass spectrometry (MS). During Co-IP, DprA, PilG, or PilE was used as bait proteins. In order to select for DprA, PilG or PilE-interacting proteins, the corresponding rabbit polyclonal antibody (Ab) against the bait protein was incubated with the cell lysates from Nm wildtype (wt) and Δ*dprA* or Δ*pilG* mutantsIn the mutants, the bait protein is absent, so the antibody will only bind the bait protein in the wt lysate. The wt bait protein coupled with the antibody binds its interacting partners and form an antibody-bait-prey protein complex. The interacting proteins were identified by MS. The "+" sign designates the formation of antibody-bait-prey protein complex whereas the "-" sign designates the absence of complex formation/interaction.

**Protein IDs**	**Protein names**	**Gene names**	**CO-IP and MS identified proteins that interact with DprA, PilG, and/or PilE**				
			Anti-DprA-Ab		Anti-PilG-Ab		Anti-PilE-Ab
			Wt	Δ*dprA*	Wt	Δ*pilG*	Wt
P05431<br>Q4W588<br>Q4W587<br>Q4W586<br>Q4W589<br>Q4W585<br>Q4W590<br>Q4W592	Fimbrial protein;PilS cassette	*pilE & pilS*	+	+	+	+	+
P0A0Q5	Holo-[acyl-carrier-protein] synthase	*acpS*	-	-	+	+	+
P0A0S6	Cell division protein FtsZ	*ftsZ*	+	+	-	-	-
P0A0S8	Ferric uptake regulation protein	*fur*	+	+	-	-	-
P0A0V3	Outer membrane protein class 4	*rmpM*	-	+	+	+	+
P0DH58	Major outer membrane protein P.IA	*porA*	+	+	+	+	+
P0DH59	Protein RecA	*recA*	-	+	+	+	-
P30690	Major outer membrane protein P.IB	*porB*	+	+	+	+	+
P42385	60 kDa chaperonin	*groL*	-	+	-	-	-
P57026	Outer membrane protein H.8	NMB1533	-	-	-	+	-
P64027	Elongation factor Tu	*tufA*	+	+	+	+	+
P64345	RNA-binding protein Hfq	*hfq*	+	+	+	+	+
P64371	Imidazoleglycerol-phosphate dehydratase	*hisB*	+	+	+	+	-
P64389	DNA-binding protein HU-beta	*hupB*	+	+	+	+	-
P65235	Ribose-phosphate pyrophosphokinase	*prs*	+	+	+	+	+

Protein IDs	Protein names	Gene names	CO-IP and MS identified proteins that interact with DprA, PilG, and/or PilE				
			Anti-DprA-Ab		Anti-PilG-Ab		Anti-PilE-Ab
			Wt	Δ*dprA*	Wt	Δ*pilG*	Wt
P66849	Single-stranded DNA-binding protein	*ssb*	+	+	+	-	+
Q4W563	PilN protein	*pilN*	+	+	+	+	+
Q70M91	Type IV pilus biogenesis and competence protein PilQ	*pilQ*	+	+	-	+	-
Q7DD45	Uncharacterized protein	NMB2094	-	+	-	+	-
Q7DD61	Uncharacterized protein	NMB1960	-	-	+	+	+
Q7DD63	Lipoprotein	NMB1946	-	-	-	+	-
Q7DD78	PilO protein	*pilO*	-	-	-	+	-
Q7DDB0	Peptidyl-prolyl cis-trans isomerase	*slyD*	+	+	-	-	-
Q7DDC4	L-lactate dehydrogenase	*lldD*	+	+	+	-	-
Q7DDI3	Class 5 outer membrane protein	*opc*	+	+	+	-	+
Q7DDJ9	2-oxoglutarate dehydrogenase, E1 component	*sucA*	-	+	-	-	-
Q7DDK4	Peroxiredoxin 2 family protein/glutaredoxin	NMB0946	+	+	-	+	-
Q7DDL0	Uncharacterized protein	NMB0830	+	-	-	-	-
Q7DDL4	Glutaredoxin	NMB0773	-	-	-	+	-
Q7DDN1	Putative iron-sulfur cluster insertion protein ErpA	*erpA*	-	-	-	+	-
Q7DDR1	Pilus assembly protein PilG	*pilG*	+	+	+	+	+
Q7DDS7	Septum site-determining protein MinD	*minD*	+	+	-	-	-
Q7DDU1	Twitching motility protein PilT	*pilT-1*	-	-	-	+	+
Q9JRV8	Acetyl-coenzyme A carboxylase carboxyl transferase subunit alpha	*accA1*	-	-	-	+	-
Q9JX95	Glyceraldehyde-3-phosphate dehydrogenase	*gapA-2*	-	+	+	+	+
Q9JXA0	Electron transfer flavoprotein, alpha subunit	*etfA*	-	+	-	+	-
Q9JXD7	Probable malate:quinone oxidoreductase	*mqo*	+	+	-	-	-
Q9JXD8	Putative adhesin complex protein	NMB2095	+	+	+	+	-
Q9JXL3	Iron-regulated outer membrane protein FrpB	*frpB*	+	-	-	-	-
Q9JXN8	Stringent starvation protein A	*sspA*	-	-	+	+	+
Q9JXP5	Probable chromosome-partitioning protein ParB	*parB*	-	-	+	+	-
Q9JXQ2	ATP synthase subunit beta	*atpD*	+	+	-	-	-
Q9JXS9	Polyphosphate kinase	*ppk*	-	-	-	+	-
Q9JXW3	Acetyl-CoA carboxylase, biotin carboxylase	*accC*	-	-	-	+	-
Q9JY02	PilM protein	*pilM*	-	+	-	-	-
Q9JY11	Uncharacterized protein	*NMB1796*	-	+	+	+	+
Q9JY16	Protein-export protein SecB	*secB*	+	+	+	+	+

Protein IDs	Protein names	Gene names	CO-IP and MS identified proteins that interact with DprA, PilG, and/or PilE				
			Anti-DprA-Ab		Anti-PilG-Ab		Anti-PilE-Ab
			Wt	Δ*dprA*	Wt	Δ*pilG*	Wt
Q9JY68	Multidrug efflux pump channel protein	*mtrE*	-	+	-	-	-
Q9JYB5	Guanylate kinase	*gmk*	-	+	-	-	-
Q9JYD2	Translation initiation factor IF-2	*infB*	-	+	+	+	-
Q9JYH3	Putative protease	NMB1587	-	-	-	+	-
Q9JYN4	Universal stress protein	NMB1500	-	-	-	+	-
Q9JYP9	Putative lipoprotein NlpD	NMB1483	-	+	+	+	-
Q9JYR2	Uncharacterized protein	NMB1468	-	-	-	+	-
Q9JYV3	Uncharacterized protein	NMB1417	+	-	-	-	-
Q9JYY0	Cysteine desulfurase IscS	*iscS*	-	+	-	-	-
Q9JYZ4	Pseudouridine synthase	NMB1361	+	-	+	+	+
Q9JZ11	Acetyltransferase component of pyruvate dehydrogenase complex	*aceF*	-	+	-	-	-
Q9JZ80	Phosphoribosylformylglycinamidine cyclo-ligase	*purM*	-	-	-	+	-
Q9JZ92	Recombination protein RecR	*recR*	+	-	-	-	-
Q9JZB5	Inosine-5-monophosphate dehydrogenase	*guaB*	+	+	+	+	-
Q9JZP6	Dihydrolipoyllysine-residue succinyltransferase component of 2-oxoglutarate dehydrogenase complex	*sucB*	+	+	-	-	+
Q9K0A4	Uncharacterized protein	NMB0711	-	+	+	+	-
Q9K0B1	Outer membrane protein assembly factor BamD	*bamD*	-	-	+	+	+
Q9K0C2	Tpc protein	*tpc*	+	+	+	-	-
Q9K0G7	UTP--glucose-1-phosphate uridylyltransferase	*galU*	-	+	-	-	-
Q9K0H1	Putative phosphate acetyltransferase Pta	NMB0631	-	+	-	+	-
Q9K0I9	Polyamine-transporting ATPase	*potA-1*	+	-	-	+	-
Q9K0P0	Alcohol dehydrogenase, propanol-preferring	*adhP*	-	+	-	+	-
Q9K0U9	Transferrin-binding protein 1	*tbp1*	+	-	-	-	-
Q9K0W0	DNA repair protein RecO	*recO*	+	-	-	-	-
Q9K0X8	Cell division protein FtsA	*ftsA*	-	+	-	-	-
Q9K0Y2	UDP-N-acetylglucosamine-N-acetylmuramyl-(pentapeptide) pyrophosphoryl-undecaprenol N-acetylglucosamine transferase	*murG*	-	-	+	+	-
Q9K147	TspA protein	NMB0341	+	+	+	-	-
Q9K177	Signal recognition particle protein	*ffh*	-	+	+	-	-

**Protein IDs**	**Protein names**	**Gene names**	**CO-IP and MS identified proteins that interact with DprA, PilG, and/or PilE**				
			Anti-DprA-Ab		Anti-PilG-Ab		Anti-PilE-Ab
			Wt	Δ*dprA*	Wt	Δ*pilG*	Wt
Q9K186	Peptidylprolyl isomerase	NMB0281	-	-	-	+	-
Q9K1D8	3-oxoacyl-[acyl-carrier-protein] synthase 2	*fabF-1*	+	+	+	+	+
Q9K1F0	Outer membrane protein assembly factor BamE	*bamE*	-	-	+	+	+
Q9K1H0	Outer membrane protein assembly factor BamA	*bamA*	-	-	+	+	-
Q9K1I8	Elongation factor G	*fusA*	-	+	-	-	-
Q9K1J1	DNA-directed RNA polymerase subunit beta	*rpoC*	+	+	-	-	-
Q9K1K0	DNA topoisomerase 1	*topA*	+	-	-	-	-
Q9K1K1	DNA processing chain A	*dprA*	+	-	-	-	-
Q9K1K7	Uncharacterized protein	NMB0109	-	+	-	+	+
